# Effect of Rivaroxaban vs Enoxaparin on Major Cardiac Adverse Events and Bleeding Risk in the Acute Phase of Acute Coronary Syndrome: The H-REPLACE Randomized Equivalence and Noninferiority Trial

**DOI:** 10.1001/jamanetworkopen.2022.55709

**Published:** 2023-02-10

**Authors:** Shenghua Zhou, Yichao Xiao, Chonglun Zhou, Zhaofen Zheng, Weihong Jiang, Qiang Shen, Can Zhu, Hongwei Pan, Changhui Liu, Gaofeng Zeng, Liangqing Ge, Yumin Zhang, Zewei Ouyang, Guang Fu, Gang Pan, Feng Chen, Lihong Huang, Qiming Liu

**Affiliations:** 1 Department of Cardiology, Second Xiangya Hospital of Central South University, Changsha, People’s Republic of China; 2 Department of Cardiology, Xiangxiang People’s Hospital, Xiangxiang, People’s Republic of China; 3 Department of Cardiology, Hunan Provincial People’s Hospital, First Affiliated Hospital of Hunan Normal University, Changsha, People’s Republic of China; 4 Department of Cardiology, Third Xiangya Hospital of Central South University, Changsha, People’s Republic of China; 5 Department of Cardiology, First People’s Hospital of Huaihua, Huaihua, People’s Republic of China; 6 Department of Cardiology, First Affiliated Hospital of Jishou University, Jishou, People’s Republic of China; 7 Department of Cardiology, First Affiliated Hospital of University of South China, Hengyang, People’s Republic of China; 8 Department of Cardiology, Second Affiliated Hospital of University of South China, Hengyang, People’s Republic of China; 9 Department of Cardiology, First People’s Hospital of Changde City, Changde, People’s Republic of China; 10 Department of Cardiology, Third Hospital of Changsha, Changsha, People’s Republic of China; 11 Department of Cardiology, Central Hospital of Shaoyang, Shaoyang, People’s Republic of China; 12 Department of Cardiology, First Hospital of Changsha, Changsha, People’s Republic of China; 13 Department of Cardiology, First People’s Hospital of Yueyang, Yueyang, People’s Republic of China; 14 Department of Biostatistics, School of Public Health, Nanjing Medical University, Nanjing, People’s Republic of China; 15 Department of Biostatistics, Zhongshan Hospital, Fudan University, Shanghai, People’s Republic of China

## Abstract

**Importance:**

Parenteral enoxaparin is a preferred anticoagulant used in the acute phase for patients with acute coronary syndrome (ACS). The safety and efficacy of short-term low-dose rivaroxaban in this clinical setting remain unknown.

**Objective:**

To compare the safety and efficacy of rivaroxaban vs enoxaparin in the acute phase of ACS.

**Design, Setting, and Participants:**

This multicenter, prospective, open-label, active-controlled, equivalence and noninferiority trial was conducted from January 2017 through May 2021 with a 6-month follow-up at 21 hospitals in China. Participants included patients with ACS missing the primary reperfusion window or before selective revascularization. Data were analyzed from November 2021 to November 2022.

**Interventions:**

Participants were randomized 1:1:1 to oral rivaroxaban 2.5 mg or 5 mg or 1 mg/kg subcutaneous enoxaparin twice daily in addition to dual antiplatelet therapy (DAPT; aspirin 100 mg and clopidogrel 75 mg once daily) for a mean of 3.7 days.

**Main Outcomes and Measures:**

The primary safety end point was bleeding events, as defined by the International Society on Thrombosis and Haemostasis, and the primary efficacy end point was major adverse cardiovascular events (MACEs), including cardiac death, myocardial infarction, rerevascularization, or stroke during the 6-month follow-up.

**Results:**

Of 2055 enrolled patients, 2046 (99.6%) completed the trial (mean [SD] age 65.8 [8.2] years, 1443 [70.5%] male) and were randomized to enoxaparin (680 patients), rivaroxaban 2.5 mg (683 patients), or rivaroxaban 5 mg (683 patients). Bleeding rates were 46 patients (6.8%) in the enoxaparin group, 32 patients (4.7%) in the rivaroxaban 2.5 mg group, and 36 patients (5.3%)in the rivaroxaban 5 mg group (rivaroxaban 2.5 mg vs enoxaparin: noninferiority hazard ratio [HR], 0.68; 95% CI, 0.43 to 1.07; *P* = .005; rivaroxaban 5 mg vs enoxaparin: noninferiority HR, 0.88; 95% CI, 0.70 to 1.09; *P* = .001). The incidence of MACEs was similar among groups, and noninferiority was reached in the rivaroxaban 5 mg group (HR, 0.60; 95% CI, 0.31 to 1.16, *P* = .02) but not in the rivaroxaban 2.5 mg group (HR, 0.68; 95% CI, 0.36 to 1.30; *P* = .05) compared with the enoxaparin group.

**Conclusions and Relevance:**

In this equivalence and noninferiority trial, oral rivaroxaban 5 mg showed noninferiority to subcutaneous enoxaparin (1 mg/kg) for patients with ACS treated with DAPT during the acute phase. Results of this feasibility study provide useful information for designing future randomized clinical trials with sufficient sample sizes.

**Trial Registration:**

ClinicalTrials.gov Identifier: NCT03363035

## Key Points

**Question:**

Is short-term oral rivaroxaban a noninferior alternative to parenteral enoxaparin in the acute phase of acute coronary syndrome (ACS)?

**Findings:**

This equivalence and noninferiority trial including 2046 patients found that short-term rivaroxaban 5 mg twice daily was noninferior to enoxaparin in terms of bleeding risk and efficacy in reducing major adverse cardiovascular events during 6 months of follow-up among patients with ACS who missed the primary reperfusion window and before selective revascularization.

**Meaning:**

These findings suggest that rivaroxaban-5 mg twice daily may be a reasonable alternative to enoxaparin in the acute phase of ACS, which may provide useful information for designing future definitive randomized clinical trials with sufficient sample sizes.

## Teaser

This randomized equivalence and noninferiority trial compares safety and efficacy of enoxaparin vs rivaroxaban 2.5 or 5 mg for patients with acute coronary syndrome (ACS) in the acute phase.

## Introduction

Patients with acute coronary syndrome (ACS) face the highest risk of death among patients with ischemic heart diseases.^1^ Primary reperfusion therapy reduced mortality of patients with ST-segment elevation myocardial infarction (STEMI) and very–high-risk non–ST-segment elevation ACS (NSTE-ACS), but nearly half of these patients may still miss the primary reperfusion therapy window, especially in developing countries.^2,3^ In addition to dual antiplatelet therapy (DAPT), parenteral enoxaparin (1 mg/kg, twice daily) is a preferred anticoagulant used in the acute phase of ACS and before selective reperfusion therapy to reduce the incidence of ischemic events^4,5,6,7,8^ and is recommended by current guidelines and routinely used in clinical practice.^9,10,11,12,13^

Rivaroxaban is a direct oral anticoagulant with both anti-Xa activity and antiplatelet effects.^14^ Recent studies indicate that rivaroxaban is noninferior to enoxaparin in preventing thromboembolism among patients with pulmonary embolism and deep venous thrombosis.^15,16^ Rivaroxaban (2.5 or 5 mg twice daily), in addition to antiplatelet therapy, is recommended for patients with stable atherosclerotic vascular disease^17^ and post-ACS.^18,19,20^

The safety and efficacy of short-term low-dose rivaroxaban in the acute phase of ACS remain unknown. To fill this evidence gap, we designed this feasibility equivalence and noninferiority trial to compare the safety and efficacy of low-dose rivaroxaban with enoxaparin in patients hospitalized with ACS, aiming to obtain primary data for the design of a definitive randomized clinical trial (RCT) with a sufficient sample size to answer this clinical question.

## Methods

This equivalence and noninferiority trial study was approved by the ethics committee of the Second Xiangya Hospital of Central South University. Approval for this trial was also obtained from the medical ethical committees at each study site, and written informed consent was obtained from all patients. The study is reported in accordance with the Consolidated Standards of Reporting Trials Extension (CONSORT Extension) reporting guideline for randomized pilot and feasibility trials.

### Study Design

In this prospective, multicenter, open-label, active-controlled, feasibility equivalence and noninferiority trial, oral rivaroxaban and subcutaneous enoxaparin were applied to patients with ACS who missed the primary reperfusion window (index events >12 or 48 hours) or before selective revascularization. The ATLAS ACS-TIMI 46 trial^19^ found that full-dose rivaroxaban plus DAPT was linked with high bleeding risk, while low-dose rivaroxaban (2.5 or 5 mg) plus DAPT reduced ischemic events, with tolerable bleeding risk. We thus opted to test the safety and efficacy of 5 mg and 2.5 mg rivaroxaban separately in our study. Details of the trial design were registered and published elsewhere^21^ and are presented in Supplement 1. The follow-up time for the primary end points was changed from 6 months to 12 months on March 2019 and changed back to 6 months on October 2020 for various reasons described in the eMethods in Supplement 2.

### Study Population

Inclusion criteria were adults aged 18 years or older; diagnosed with ACS (STEMI, non-ST segment elevation myocardial infarction [NSTEMI], or unstable angina); patients with STEMI who missed the recommended time window for revascularization (index events >12 hours without ongoing symptoms suggestive of ischemia, hemodynamic instability, or life-threatening arrhythmias; or asymptomatic patients with index events >48 hours),^11^ or patients with NSTE-ACS without an indication for primary percutaneous coronary intervention (PCI) during the next 24 hours and waiting for selective revascularization^12^; and indication for short-term use of enoxaparin combined with DAPT. Exclusion criteria were patients who already received thrombolytic therapy or revascularization or revascularization therapy was planned within 12 hours, patients who received glycoprotein IIb/IIIa inhibitors, patients with increased bleeding risk or severe concomitant disease (eAppendix 1 in Supplement 2), patients with indications for long-term oral anticoagulation therapy (such as atrial fibrillation, venous thromboembolism, or prior mechanical heart valves replacement), patients with contraindications for rivaroxaban or enoxaparin, and patients enrolled in another clinical study.

### Study Randomization and Treatment Regimens

Eligible participants were randomized in a 1:1:1 ratio with a simple randomization method using an interactive web response system to the 3 intervention groups. Participants received either oral rivaroxaban 2.5 mg, rivaroxaban 5 mg, or subcutaneous enoxaparin (1 mg/kg) twice daily until discharge or 12 hours before revascularization therapy for up to 8 days. All participants received DAPT (100 mg aspirin and 75 mg clopidogrel once daily), and DAPT was continued when indicated after discharge. The protocol of periprocedural antithrombotic management is summarized in eAppendix 2 in Supplement 2.

### Study End Points

There were 2 primary end points in this study. The primary safety end point was a composite of major bleeding, clinically relevant nonmajor bleeding, and minor bleeding events, as defined by the International Society on Thrombosis and Haemostasis (ISTH). The primary efficacy end point was major adverse cardiovascular events (MACEs), a composite end point of cardiac death, myocardial infarction, re-revascularization, or stroke during the 6-month follow-up period. The secondary end points included all-cause death and cardiac-related rehospitalization. Complete definitions of the end points are provided in eAppendix 3 in Supplement 2. All clinical events were reviewed, classified, and evaluated by an independent adjudication committee whose members were unaware of the study group assignments (eAppendix 4 in Supplement 2).

### Statistical Analysis

As a feasibility trial, approximately 2000 patients were expected to be enrolled during the study period. For feasible noninferiority purposes, the noninferior margin (Δ) for the primary efficacy end point was set at 1.185 according to a previous publication.^22^ The noninferior margin for the primary safety end point was set at 1.24. For this margin, we estimated the potential margin according to the fix-margin approach recommended by the US Food and Drug Administration (FDA)^23^ based on the parameter regarding major bleeding events from a meta-analysis,^24^ and the common margin of (0.8-1.25) in the bio-equivalency study.^25^ The detailed calculations are described in eAppendix 5 in Supplement 2.

Sequential noninferiority analyses were used for the primary end points to explore the feasibility of the coprimary end points and fixed-sequence testing procedure. The end points were analyzed using a fixed-sequence testing procedure in order of (1) safety end point between the rivaroxaban 2.5 mg and enoxaparin groups, (2) safety end point between the rivaroxaban 5 mg and enoxaparin groups, (3) efficacy end point between the rivaroxaban 5 mg and enoxaparin groups, and (4) efficacy end point between the rivaroxaban 2.5 mg and enoxaparin groups. The noninferiority analysis was to be followed by a superiority analysis if noninferiority analysis was concluded.

Baseline demographic characteristics, including self-reported race and ethnicity (categorized by Han, Tujia, Miao, and other minority [eg, Hui, Mongol]) were collected prior to randomization. We collected this information to determine whether the racial and ethnic backgrounds of study participants were similar among groups. The baseline characteristics are presented as the mean and SD for continuous variables and frequency and percentage for categorical variables. Survival analyses were based on the time to the first event. The cumulative incidence of the time-to-event end points is expressed as Kaplan-Meier curves over 6 months after randomization. Cox proportional hazard models were used to estimate hazard ratios (HRs) and 2-sided 95% CIs. The *P* value for noninferiority analysis was calculated following published methods.^26^ In addition, we performed an analysis of early and late outcomes using the in-hospital and 30-day time points as landmarks. Subgroup analyses were performed based on several major risk factors, including age, sex, body mass index, creatinine clearance, smoking, medical history (hypertension, dyslipidemia, diabetes, previous myocardial infarction, PCI or coronary artery bypass graft [CABG], or stroke), and index diagnosis (STEMI, NSTEMI, and unstable angina). The noninferiority analysis of safety and efficacy was performed using a per-protocol population, and an intention-to-treat (ITT) population was applied for sensitivity analyses.

The noninferiority analyses for primary end points were sequential, and 1-sided α = .025 was applied for each successive step. For the other analyses, the common 2-sided tests with α = .05 were used. All statistical analyses were conducted using SAS software version 9.4 (SAS Institute). Data were analyzed from November 2021 to November 2022.

## Results

### Baseline Characteristics of Study Participants

**Figure 1.**
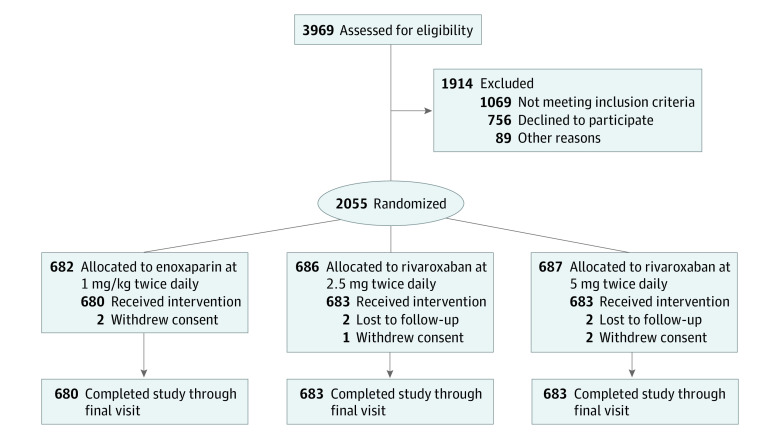
Flowchart of Trial Participation

From January 2017 through November 2020, 2055 patients were enrolled at 21 study centers. Four patients were lost to follow-up (2 patients in the rivaroxaban 2.5 group and 2 patients in the rivaroxaban 5 mg group), 5 patients decided to withdraw from the study after randomization for individual reasons before receiving rivaroxaban or enoxaparin, and 2046 patients (mean [SD] age 65.8 [8.2] years, 1443 [70.5%] male) were randomized to enoxaparin (680 patients), rivaroxaban 2.5 mg (683 patients), or rivaroxaban 5 mg (683 patients) and finished the 6-month follow-up (Figure 1). Baseline characteristics were similar among groups (Table 1). The mean (SD) duration of the study drug was similar for each arm (enoxaparin: 3.6 [1.3] days; rivaroxaban 2.5 mg: 3.7 [1.1] days; rivaroxaban 5 mg: 3.9 [1.0] days). A total of 1455 patients (71.1%) received reperfusion therapy (PCI or CABG) for the index diagnosis before discharge.

**Table 1.** Baseline Characteristics of Study Participants^a^

Characteristic^a^	Patients, No. (%)		
	Enoxaparin, 1 mg/kg (n = 680)^a^	Rivaroxaban	
		2.5 mg (n = 683)	5 mg (n = 683)
Age, mean (SD), y	65.3 (8.7)	66.2 (7.8)	65.8 (8.2)
Sex			
Female	196 (28.8)	200 (29.3)	207 (30.3)
Male	484 (71.2)	483 (70.7)	476 (69.7)
Race and ethnicity			
Han	614 (90.3)	622 (91.1)	616 (90.2)
Tujia	41 (6.0)	389 (5.6)	40 (5.8)
Miao	22 (3.2)	21 (3.1)	25 (3.7)
Other^b^	3 (0.4)	2 (0.3)	2 (0.3)
BMI, mean (SD)	22.84 (3.97)	23.23 (4.09)	22.26 (4.09)
Creatinine clearance, median (IQR), mL/min^c^	84.1 (67.4-104.8)	85.2 (68.1-106.2)	83.2 (63.1-106.4)
Smoking	161 (23.7)	160 (23.4)	158 (23.1)
Medical history			
Hypertension	433 (63.7)	423 (61.9)	417 (61.1)
Dyslipidemia	403 (59.3)	398 (58.3)	384 (56.2)
Diabetes	231 (34.0)	215 (31.5)	231 (33.8)
Previous myocardial infarction	110 (16.2)	98 (14.3)	97 (14.2)
Previous PCI or CABG	149 (21.9)	152 (22.3)	141 (20.6)
Previous stroke	39 (5.7)	36 (5.3)	35 (5.1)
Index diagnosis			
STEMI	284 (41.8)	259 (37.9)	269 (39.4)
NSTEMI	199 (29.2)	227 (33.2)	218 (31.9)
Unstable angina	197 (29.0)	197 (28.9)	196 (28.7)
PCI or CABG for index event			
Any	484 (71.0)	490 (71.7)	481 (70.4)
PCI	475 (69.9)	484 (70.8)	473 (69.3)
CABG	9 (1.3)	6 (0.9)	8 (1.2)
Medication use			
β-blocker	421 (61.9)	419 (61.3)	439 (64.3)
ACE inhibitor or ARB	293 (43.1)	302 (44.2)	307 (44.9)
Statin	569 (83.7)	592 (86.7)	561 (82.1)
Calcium-channel block	147 (21.6)	126 (18.4)	161 (23.6)
Procedural aspects			
Radial access	467 (98.3)	470 (97.1)	463 (97.9)
Clot burden (TIMI thrombus grade ≥3)	87 (18.3)	102 (21.1)	91 (19.2)
TIMI flow grade			
0	44 (9.3)	52 (10.7)	46 (9.7)
1	12 (2.5)	21 (4.3)	22 (4.7)
2	31 (6.5)	29 (6.0)	23 (4.9)
3	388 (81.7)	382 (78.9)	382 (80.8)
Duration of anticoagulation, mean (SD), min	52.6 (11.2)	55.9 (10.8)	53.5 (12.1)
Reasons for missing opportunity of direct reperfusion			
Patient delay	173 (60.9)	158 (61.0)	179 (66.5)
Financial issues	43 (15.1)	48 (18.5)	39 (14.5)
Misdiagnosis	21 (7.4)	17 (6.5)	22 (8.2)
Others	47 (16.5)	36 (13.9)	29 (10.8)

Abbreviations: ACE, angiotensin-converting enzyme; ARB, angiotensin-receptor blocker; BMI, body mass index (calculated as weight in kilograms divided by height in meters squared); CABG, coronary artery bypass grafting; NSTEMI, non-ST segment elevation myocardial infarction; PCI, percutaneous coronary intervention; STEMI, ST-segment elevation myocardial infarction; TIMI, Thrombolysis in Myocardial Infarction.

^a^ There were no significant differences among the 3 groups.

^b^ Other minority ethnic groups included Hui and Mongol.

^c^ Creatinine clearance was calculated with the Cockcroft–Gault equation.

### Primary Safety End Points

**Figure 2.**
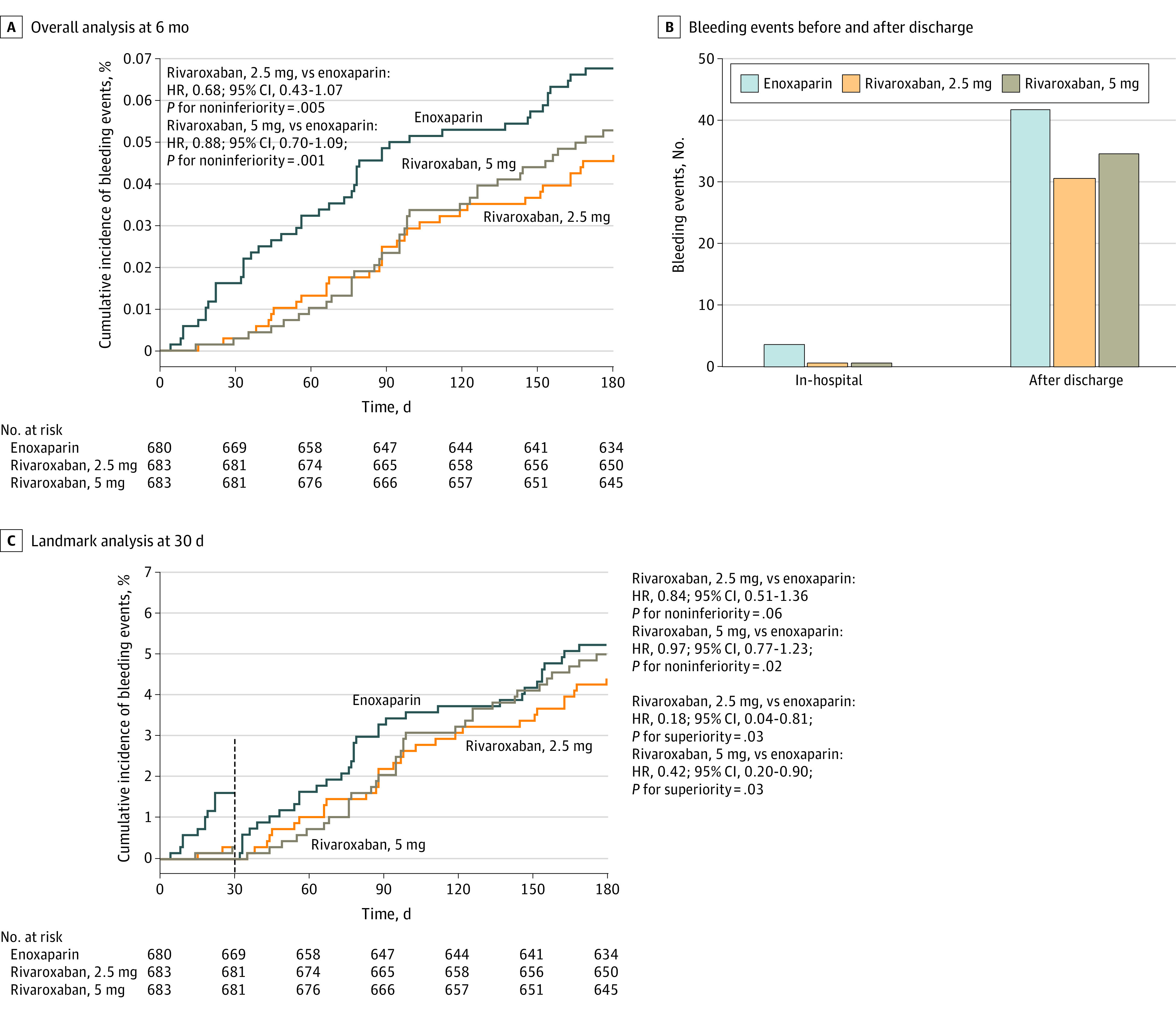
Cumulative Incidence of Bleeding Events The definition of bleeding is provided in eTable 1 in Supplement 2. HR indicates hazard ratio.

A total of 114 bleeding events (5.6%) occurred, including 46 events (6.8%) in the enoxaparin group, 32 (4.7%) in the rivaroxaban 2.5 mg group, and 36 (5.3%) in the rivaroxaban 5 mg group. After 6 months of follow-up, noninferiority of rivaroxaban vs enoxaparin was reached for primary safety end points, in that the upper boundary of the 95% CI of the HR in bleeding events was below the predefined margin (rivaroxaban 2.5 mg: HR, 0.68; 95% CI, 0.43-1.07; *P* = .005; rivaroxaban 5 mg: HR, 0.88; 95% CI, 0.70-1.09; *P* = .001) (Figure 2A).

### Primary Efficacy End Point

**Figure 3.**
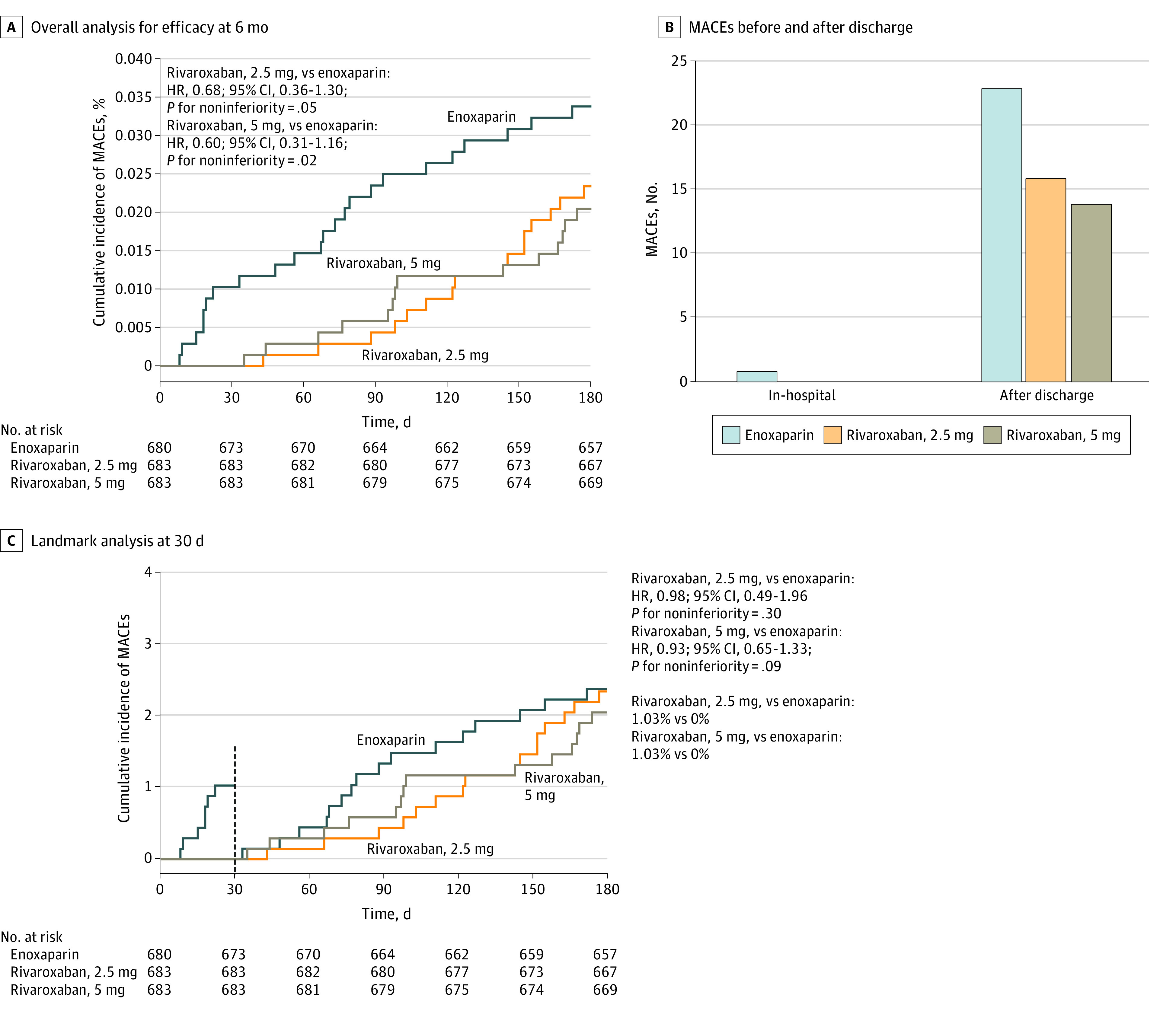
Cumulative Incidence of Primary Efficacy End Points The primary efficacy end points was a composite of cardiac death, myocardial infarction, re-revascularization, and stroke. HR indicates hazard ratio; MACE, major adverse cardiovascular event.

MACEs occurred in 14 patients (2.1%) in the rivaroxaban 5 mg group and in 23 patients (3.4%) in the enoxaparin group (noninferiority: HR, 0.60; 95% CI, 0.31-1.19, *P* = .02). MACEs were observed in 16 patients (2.3%) in the rivaroxaban 2.5 mg group and did not reach noninferiority (noninferiority: HR, 0.68; 95% CI, 0.36-1.30; *P* = .05) (Table 2 and Figure 3A).

**Table 2.** Incidence of the Primary and Secondary End Points

End points	No. (%)			Rivaroxaban 2.5 mg vs Enoxaparin		Rivaroxaban 5 mg vs Enoxaparin	
	Enoxaparin, 1 mg/kg (n = 680)	Rivaroxaban					
		2.5 mg (n = 683)	5 mg (n = 683)	HR (95% CI)	*P* value^a^	HR (95% CI)	*P* value^a^
**Safety**							
Any bleeding^b^	46 (6.8)	32 (4.7)	36 (5.3)	0.68 (0.43-1.07)	.005^b^	0.88 (0.70-1.09)	.001^b^
ISTH							
Major bleeding	8 (1.2)	5 (0.7)	5 (0.7)	0.62 (0.20-1.90)	NA	0.62 (0.20-1.90)	NA
CRNM bleeding	9 (1.3)	8 (1.2)	8 (1.2)	0.89 (0.34-2.29)	NA	0.88 (0.34-2.28)	NA
Minor bleeding	29 (4.3)	19 (2.8)	23 (3.4)	0.68 (0.43-1.07)	NA	0.77 (0.50-1.19)	NA
**Efficacy**							
Composite end point^b^	23 (3.4)	16 (2.3)	14 (2.1)	0.68 (0.36-1.30)	.05^b^	0.60 (0.31-1.16)	.02^b^
Cardiac death	3 (0.4)	2 (0.3)	2 (0.3)	0.66 (0.11-3.98)	NA	0.66 (0.11-3.97)	NA
Myocardial infarction	9 (1.3)	6 (0.9)	5 (0.7)	0.66 (0.24-1.86)	NA	0.55 (0.18-1.64)	NA
Re-revascularization	3 (0.4)	2 (0.3)	2 (0.3)	0.66 (0.11-3.97)	NA	0.66 (0.11-3.97)	NA
Stroke							
Any	8 (1.2)	6 (0.9)	5 (0.7)	0.74 (0.26-2.14)	NA	0.62 (0.20-1.90)	NA
Ischemic	4 (0.6)	2 (0.3)	3 (0.4)	0.50 (0.09-2.71)	NA	0.74 (0.17-3.33)	NA
**Secondary efficacy end points**							
All-cause death	5 (0.7)	3 (0.4)	4 (0.6)	0.60 (0.14-2.50)	NA	0.80 (0.21-2.97)	NA
Cardiac-related rehospitalization	56 (8.7)	49 (7.2)	51 (7.5)	0.85 (0.58-1.25)	NA	0.89 (0.61-1.30)	NA

Abbreviations: CRNM, clinically relevant nonmajor; HR, hazard ratio; ISTH, International Society on Thrombosis and Haemostasis; NA, not applicable.

^a^ *P* values for noninferiority were calculated by PROBNORM ([estimate-LN(L)]/SE), where PROBNORM is the standard normal distribution function, estimate = Parameter estimate, L = Margin.

^b^ Primary end points were tested sequentially from any bleeding in rivaroxaban 2.5 mg vs enoxaparin to any bleeding in rivaroxaban 5 mg vs enoxaparin to the composite efficacy end point (cardiac death, myocardial infarction, re-revascularization, or stroke) in rivaroxaban 5 mg vs enoxaparin to the composite efficacy end point in rivaroxaban 2.5 mg vs enoxaparin.

### Secondary End Points and Other Analyses

The incidence of ISTH major bleeding events was similar among groups. No fatal bleeding events were observed. The relative risk of death from any cause and cardiac-related rehospitalization was similarly low in both rivaroxaban groups and the enoxaparin group (Table 2). All end points were also analyzed based on the ITT population, and the results were consistent with those in the per-protocol population (eTable 2 and eTable 3 in Supplement 2).

Landmark survival analysis of early and late events was also performed with 2 landmark time points (in-hospital and 30 days after randomization). Bleeding events or MACEs during hospitalization were rare and numerically lower in both low-dose oral rivaroxaban groups than in the enoxaparin group (Figure 2B and Figure 3B). Landmark analysis for the primary end point at 30 days after randomization found significantly lower bleeding events in the rivaroxaban 5 mg group (HR, 0.42; 95% CI, 0.20 to 0.90; *P* = .03) and rivaroxaban 2.5 mg group (HR, 0.18; 95% CI, 0.04 to 0.81; *P* = .03) than in the enoxaparin group (Figure 2C and Figure 3C). No MACEs occurred in either low-dose rivaroxaban group, while 7 events occurred in the enoxaparin group. Late events analysis for the primary end point showed safety noninferiority was still reached in the rivaroxaban 5 mg group (noninferiority: HR, 0.97; 95% CI, 0.77 to 1.23; *P* = .02). The analysis results of most subgroups (eg, sex, body mass index, creatinine clearance, smoking) were found to be essentially consistent with the overall results (eFigure 1 and eFigure 2 in Supplement 2).

## Discussion

The main finding of this equivalence and noninferiority feasibility trial is that rivaroxaban 5 mg twice daily in addition to DAPT in the acute phase of ACS for patients who missed the primary reperfusion window or before selective revascularization therapy was noninferior to enoxaparin in terms of bleeding risk and efficacy during the 6-month follow-up period. This finding provides useful information for designing future definitive RCTs with sufficient sample sizes to verify this clinical question. To our knowledge, this is the first clinical equivalence and noninferiority study describing the potential safety and efficacy of short-term low-dose rivaroxaban use in the acute phase of ACS.

The noninferior margin we set is clinically accepted based on results of previous studies and FDA guidelines. The actual incidence of efficacy and safety end points may be used for calculating the sample size for future definitive RCTs.

For patients with ACS missing the primary reperfusion therapy window, anticoagulation is effective in reducing ischemic events with background DAPT.^9,10^ The patients included in this study were similar to patients included in the TETAMI trial.^9^ However, patients with NSTE-ACS before selective invasive strategy (mainly referring to NSTE-ACS at intermediate or low risk^11^) were also enrolled in our study.

Rivaroxaban exerts independent antiplatelet effects in addition to potent anticoagulant effects.^27^ Several previous trials^18,19,20,28,29^ explored the effects of adding oral anticoagulant to DAPT in patients with ACS. The ATLAS ACS 2-TIMI 51 study^18^ showed that rivaroxaban (2.5 mg twice daily) plus DAPT for a mean of 13 months reduced the incidence of MACEs in patients with stabilized ACS. Our study found that short-term rivaroxaban 5 mg plus DAPT in the acute phase was noninferior to enoxaparin plus DAPT in both the primary safety and efficacy end points during the 6-month follow-up period for patients with ACS. Moreover, the MACEs rate was lower in the rivaroxaban 5 mg plus DAPT group than in the enoxaparin plus DAPT group at 6 months after randomization. The observed effects could be partly explained by the dual antiplatelet and anticoagulant effects of rivaroxaban. The incidence of ischemic events reported in this study was lower than that reported in patients treated with a loading dose of enoxaparin. Several factors might be responsible for the lower ischemic events: the relatively small cohort size that also only enrolled individuals of a single racial and ethnic group and exclusion of patients with ACS requiring primary PCI, who were usually patients with high-risk ACS. Most of the enrolled patients received selective PCI during hospitalization.

Our objective was to explore whether rivaroxaban can be a substitute for enoxaparin during the acute phase of ACS. Therefore, rivaroxaban was not continued after the study period. The GEMINI-ACS-1 trial^20^ demonstrated similar effects of rivaroxaban 2.5 mg twice daily vs aspirin 100 mg daily in addition to a P2Y_12_ inhibitor for patients with ACS started within 10 days after index diagnosis and continued for 6 to 12 months. We are unable to speculate whether continuation of our therapy regimen could result in more favorable clinical outcomes. Future studies are warranted to explore this issue. It should be noted that observing the impact of the short-term treatment on the clinical outcome at 6 months is challenging, and bias is inevitable. The effect of short-term medication on outcome at 6 months was previously explored in the RELAX-AHF trial.^30^ The study showed that standard care plus 48-hour intravenous infusions of serelaxin resulted in significant reductions in other prespecified additional end points, including fewer deaths at day 180. The landmark analysis demonstrated the noninferiority of low-dose rivaroxaban to enoxaparin within 30 days in our study.

As a feasibility trial with an insufficient sample size, our study supplied some useful information for designing future large-scale RCTs to verify our results, especially the appropriate noninferior margin for the primary end point of future definitive RCTs and the potential safety and that the efficacy of low-dose of rivaroxaban in patients hospitalized with ACS was clinically acceptable.

Rivaroxaban has the advantages of being an oral therapy and fixed dose for all patients. Thus, rivaroxaban may be a good choice for prehospital management and may improve medication adherence. Our findings may help clinicians and patients with ACS in the acute phase choose the optimal anticoagulant therapy during the decision-making process.

Although rivaroxaban at a dose of 2.5 mg did not show statistical noninferiority to enoxaparin in terms of the efficacy outcome, the MACE rate at the 6 months was similarly low in rivaroxaban 2.5 mg and enoxaparin groups. Therefore, patients with ACS may still have a chance to choose either a 2.5 or 5 mg twice daily dose plan according to their individual bleeding risk. Future regular noninferiority trials with larger sample sizes would fully address this gap.

### Limitations

There are several limitations to address. First, although our results may provide a reference for anticoagulant care in patients with ACS in the acute phase, all patients included in this study were Chinese, which could limit the generalizability of the findings to patients of different nationalities and races. Further studies with other cohorts of patients with ACS of different nationalities and races are needed. Second, this study was a feasibility randomized equivalence and noninferiority trial to demonstrate the potential safety and efficacy of rivaroxaban vs enoxaparin in patients with ACS. Although some end point events between groups reached noninferiority, the power was low due to small sample size. A planned future trial with a sample size of 5655 for each group will aim to address these questions. Third, the use of a 6-month time window in noninferiority assessment of safety and efficacy of an antithrombotic trial regimen given for the first 4 days only may increase the false-positive rate (α error) in the trial, as the addition of events that do not bear on the randomized treatment may increase the power of the trial. However, future trials may select primary end points in a short-term follow-up (eg, 1 month), while the sample size may increase exponentially with a lower incidence of events. Fourth, the use of an open-label study may have introduced some degree of bias related to the patients and physicians not being blinded to the assigned treatment. We attempted to minimize any potential bias by having all clinical events reviewed and adjudicated by an independent clinical events committee. Fifth, although the use of ticagrelor or prasugrel is not currently recommended as part of triple antithrombotic therapy with aspirin and oral anticoagulant,^31,32^ the efficacy and safety of anticoagulation when combined with these more potent P2Y_12_ inhibitors remain unclear. Sixth, minor bleeding was reported by the investigators and not adjudicated, as were the cases of clinically relevant nonmajor or major bleeding. In turn, underreporting is possible, and this point should be kept in mind when interpreting the effect estimates for minor bleeding events. Seventh, although randomization is helpful to exclude the interference of other cofounders on the outcome, the potential for bias cannot be excluded. The inclusion of all bleeding events during the 6 months in the primary safety end point might create bias (or push the data) toward the (alternative) hypothesis of noninferiority of rivaroxaban in either dosage vis-à-vis enoxaparin. The addition of these later bleeding events, equally distributed among the groups, might potentially increase the power of the trial.

## Conclusions

In this randomized, controlled, equivalence and noninferiority trial of patients with ACS in the acute phase, the use of short-term rivaroxaban (5 mg twice daily) was noninferior to enoxaparin in terms of both safety and efficacy outcomes in addition to DAPT. The data obtained from this small trial might be helpful for designing future noninferiority trials with sufficient sample sizes to test whether rivaroxaban 5 mg twice daily plus DAPT might be a reasonable alternative regimen to enoxaparin plus DAPT during the acute phase of ACS for patients who missed the primary reperfusion window or not.

## Notes

Supplement 1.
Trial Protocol and Statistical Analysis Plan

Supplement 2. **eMethods.** Protocol Amendment **eAppendix 1.** Inclusion and Exclusion Criteria **eAppendix 2.** Periprocedural Antithrombotic Management **eAppendix 3.** End Point Definitions **eAppendix 4.** Standard Operation Procedure for Adjudication Process **eAppendix 5.** Determination of Noninferior Margin **eTable 1.** Definitions of ISTH Bleeding Events **eTable 2.** Baseline Characteristics (ITT Analysis) **eTable 3.** Primary and Secondary End Points (ITT Analysis) **eFigure 1.** Risk of Primary Safety End Point Based on Major Subgroup **eFigure 2.** Risk of Primary Efficacy End Point Based on Major Subgroup
eReferences.

Supplement 3.
The H-REPLACE Investigators

Supplement 4.
Data Sharing Statement
