# Supplementary material for: Effect of Rivaroxaban vs Enoxaparin on Major Cardiac Adverse Events and Bleeding Risk in the Acute Phase of Acute Coronary Syndrome: The H-REPLACE Randomized Equivalence and Noninferiority Trial
Source: JAMA Netw Open. 2023 Feb 10;6(2):e2255709. doi: 10.1001/jamanetworkopen.2022.55709 (PMC9918885; doi:10.1001/jamanetworkopen.2022.55709)
Supplement: Supplement 1. — Trial Protocol and Statistical Analysis Plan [file jamanetwopen-e2255709-s001.pdf]

Safety and efficacy of low-dose rivaroxaban in the acute phase of acute coronary syndrome: an open-label, randomized, noninferiority comparison (H-REPLACE)

Study protocol

Research Initiating Unit: Second Xiangya Hospital of Central South University

Type of study: Prospective, multicenter, randomized, controlled study

Principal investigator: Shenghua Zhou

Project ID: H-REPLACE-201711

Vision: Version 4.0

Date: October 10, 2020

TABLE OF CONTENTS

TITLE PAGE **1**

TABLE OF CONTENTS **2**

SYNOPSIS **3**

1. BACKGROUND **4**

2. STUDY RATIONALE **6**

3. OBJECTIVES **7**

4. STUDY PROTOCOL **8**

4.1 STUDY DESIGN AND DURATION 8
4.2 POPULATION 8
4.3 INFORMED CONSENT WAS SIGNED. 10
4.4 RANDOMIZATION 11
4.5 TREATMENT 11
4.6 STUDY ENDPOINT 13

5. STUDY ASSESSMENTS AND PROCEDURES **15**

5.1 COLLECTING DATA 15
5.2 STANDARD OPERATING PROCEDURES FOR CLINICAL STUDY DATA RECORDING: 17
5.3 TRIAL MANAGEMENT 18
5.4 PATIENT ADHERENCE MANAGEMENT 19
5.5 SUBJECT COMPLETION/WITHDRAW 20
5.6 DATA MANAGEMENT 21

6. STATISTICAL METHOD **22**

6.1 NONINFERIOR MARGIN 22
6.2 SAMPLE SIZE ESTIMATION 22
6.3 STATISTICAL ANALYSIS 23

7. RESEARCH MANAGEMENT **24**

7.1 RESEARCH INSTITUTIONS 24
7.2 MONITORING AND AUDITING 25
7.3 MANAGEMENT AND PRESERVATION OF FILES 25
7.4 CONSENT PROCEDURE AND CHANGE PROCEDURE OF THE EXPERIMENTAL PROTOCOL 25

8. ETHICAL REQUIREMENTS **26**

9. PROTOCOL MODIFICATIONS **26**

10. REFERENCES **28**

SYNOPSIS

Acute coronary syndrome (ACS) is a serious and life-threatening condition. Anticoagulation during the acute phase of ACS is effective in reducing ischemic events. The most widely used parenteral anticoagulation agent in ACS patients is enoxaparin. Rivaroxaban is a novel oral anticoagulant with potent anti-Xa activity, which might be an attractive alternative drug to enoxaparin. In fact, rivaroxaban was consistently shown to be noninferior to enoxaparin therapy in terms of the reduction of recurrent venous thromboembolism events. This prospective, multicenter, open-label, randomized, active-controlled, noninferiority feasibility study is designed to compare the safety and efficacy of rivaroxaban versus enoxaparin in patients with ACS who missed the primary reperfusion therapy window and before selective revascularization. Participants receiving background treatment of aspirin plus clopidogrel or ticagrelor will be randomly assigned to either oral rivaroxaban 2.5 mg twice daily or rivaroxaban 5 mg twice daily or subcutaneous enoxaparin 1 mg/kg twice daily until hospital discharge for a maximum of 8 days or 12 h before revascularization therapy. The primary safety endpoint is the International Society on Thrombosis and Hemostasis definition of bleeding events [minor, clinically relevant nonmajor and major bleeding]. The primary efficacy endpoint is a composite of major adverse cardiac events (MACEs), including cardiac death, myocardial infarction, rerevascularization or stroke, and major bleeding events. Secondary endpoints include cardiac-related rehospitalization and all-cause death. Patients will be followed for 6

months after randomization.

1. Background

1.1 Acute coronary syndrome (ACS) seriously endangers public health

Cardiovascular disease death accounts for the first place in the total deaths of urban and rural residents in China, with 45% in rural areas and 43% in urban areas; coronary heart disease is an important cause of death, and acute coronary syndrome (ACS) is the main cause of coronary heart disease. Types of. ACS is a group of clinical syndromes caused by acute myocardial ischemia, including unstable angina (UA), ST-segment elevation myocardial infarction (STEMI), and non-ST-segment elevation myocardial infarction (NSTEMI). ACS is characterized by acute onset, rapid changes in condition, and poor prognosis. The hospitalized and long-term mortality rates are 6% and 12%, respectively [1], which seriously endangers social and public health.

1.2 ACS anticoagulation and antiplatelet therapy are equally important, and the guidelines recommend the short-term combined use of LMWH+DAPT

Regardless of the subtype of ACS, thrombosis and/or thromboembolism are the predominant pathophysiological mechanisms. Therefore, anticoagulation drugs play an extremely important role in the treatment of ACS. Platelet activation and coagulation system activation are two crucial links in the process of thrombosis and/or thromboembolism; the two are closely linked in the body, and thrombin generated after coagulation system activation is a powerful platelet activating factor. Activation of platelets in turn promotes the coagulation process. Antithrombotic

therapy should target both the coagulation system (anticoagulation therapy) and platelets (antiplatelet therapy). DAPT therapy is the cornerstone of the treatment of ACS patients and has received enough attention in clinical practice, while anticoagulation therapy is often neglected. Based on evidence from a large number of high-quality studies, guidelines[2,3] clearly recommend that short-term (less than 8 days) combined use of anticoagulant and antiplatelet drugs for ACS can reduce the incidence of thrombotic events and improve prognosis in patients with ACS. Enoxaparin is a low molecular weight heparin with the strongest anticoagulant effect. The short-term combined use of enoxaparin anticoagulation and aspirin + P2Y12 receptor antagonist dual antiplatelet (DAPT) is a class IA recommendation for patients with UA, NSTEMI, and STEMI during conservative drug treatment or waiting for PCI.

1.3 The new oral anticoagulant rivaroxaban has obvious advantages over enoxaparin

The peak time and half-life of rivaroxaban are superior or noninferior to enoxaparin[4-6], and rivaroxaban is an oral drug and does not require subcutaneous injection; the dose is fixed and does not need to be adjusted by body weight. Can increase compliance. Clinical studies related to anticoagulation in patients with pulmonary embolism/deep vein thrombosis and hip/knee replacement [7-9] have confirmed that the safety and efficacy of rivaroxaban are superior or noninferior to enoxaparin.

1.4 Safety of short-term rivaroxaban combined with enoxaparin

The ATLAS ACS TIMI 46 study [10] showed that the incidence of clinically

significant bleeding (approximately 1.5%-2.5%) in 2.5 mg BID and 5 mg BID in the short term (8 days) with rivaroxaban combined with DAPT was much lower than that of enoxaparin. The incidence of bleeding in combination with DAPT is 3% to 9% [11].

In conclusion, we hypothesized that the safety and efficacy of short-term anticoagulation with rivaroxaban during hospitalization in ACS patients treated with DAPT is noninferior to enoxaparin.

2. Study rationale

This study is a prospective, multicenter, open-label, randomized, active-controlled, noninferiority feasibility study.

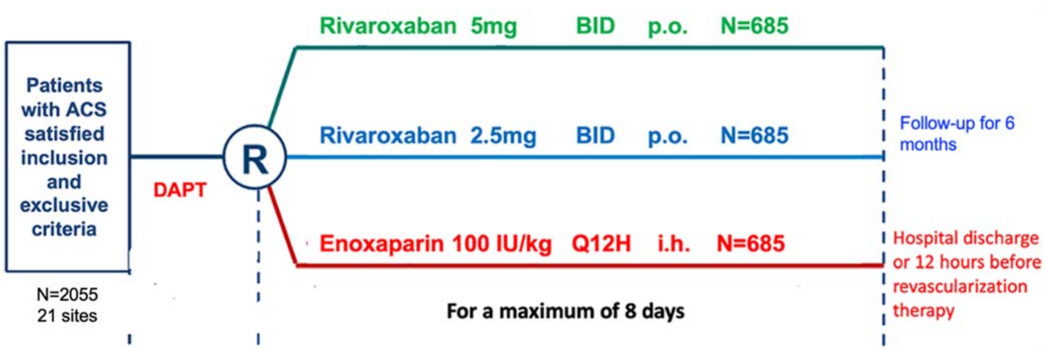

This feasibility trial compared the safety and efficacy of rivaroxaban versus enoxaparin in patients with ACS who missed the primary reperfusion therapy window and before selective revascularization. Participants receiving background treatment of aspirin plus clopidogrel or ticagrelor will be randomly assigned to either oral rivaroxaban 2.5 mg twice daily or rivaroxaban 5 mg twice daily or subcutaneous

enoxaparin 1 mg/kg twice daily until hospital discharge for a maximum of 8 days or 12 h before revascularization therapy. The primary safety endpoint is the International Society on Thrombosis and Hemostasis definition of bleeding events [minor, clinically relevant nonmajor and major bleeding]. The primary efficacy endpoint is a composite of major adverse cardiac events (MACEs), including cardiac death, myocardial infarction, rerevascularization or stroke, and major bleeding events. Secondary endpoints include cardiac-related rehospitalization and all-cause death. Patients will be followed for 6 months after randomization.

3. Objectives

To compare the safety and efficacy of rivaroxaban versus enoxaparin in patients with ACS who missed the primary reperfusion therapy window and before selective revascularization.

3.1 Primary objective

1) Is the bleeding risk (ISTH bleeding) of short-term in-hospital low-dose rivaroxaban in ACS patients noninferior to low-molecular-weight heparin?

2) Is the risk of cardiovascular events (cardiac death, myocardial infarction (including reinfarction), stroke (ischemic, hemorrhagic and unexplained) and the need for repeat blood in ACS patients with short-term low-dose rivaroxaban in hospital noninferior to low molecular weight heparin?

3.2 Secondary objective

1) Can short-term in-hospital low-dose rivaroxaban in ACS patients reduce

psychogenic hospitalization compared with low-molecular-weight heparin?

2) Can short-term in-hospital application of low-dose rivaroxaban in ACS patients reduce all-cause mortality compared with low-molecular-weight heparin?

4. Study protocol

4.1 Study design and duration

The H-REPLACE study is a prospective, multicenter, open-label, randomized, active-controlled, noninferiority feasibility study which is designed to compare the safety and efficacy of rivaroxaban versus enoxaparin in patients with ACS who missed the primary reperfusion therapy window and before selective revascularization. Participants receiving background treatment of aspirin plus clopidogrel or ticagrelor will be randomly assigned to either oral rivaroxaban 2.5 mg twice daily or rivaroxaban 5 mg twice daily or subcutaneous enoxaparin 1 mg/kg twice daily until hospital discharge for a maximum of 8 days or 12 h before revascularization therapy. The primary safety endpoint is the International Society on Thrombosis and Hemostasis definition of bleeding events [minor, clinically relevant nonmajor and major bleeding]. The primary efficacy endpoint is a composite of major adverse cardiac events (MACEs), including cardiac death, myocardial infarction, rerevascularization or stroke, and major bleeding events. Secondary endpoints include cardiac-related rehospitalization and all-cause death. Patients will be followed for 6 months after randomization.

4.2 Population

Inclusion and exclusion criteria of the H-REPLACE trial.

Inclusion criteria

- Male or female aged more than 18 years
- Diagnosed with ACS (STEMI, NSTEMI, unstable angina) who missed primary reperfusion window and before selective revascularization
- With an indication for short-term combination use of DAPT and enoxaparin.

Exclusion criteria

- Already received thrombolytic therapy or revascularization or needing revascularization therapy in 12 hours.
- With platelet glycoprotein IIb/IIIa receptor antagonist therapy.
- With increased bleeding risk, such as but not limited to, active internal bleeding, clinically significant bleeding, bleeding at a noncompressible site, or bleeding diathesis within 30 days of randomization; platelet count less than 90,000/μL at screening; intracranial hemorrhage; major surgery, biopsy of a parenchymal organ, or serious trauma within 30 days before randomization; clinically significant gastrointestinal bleeding within 12 months before randomization; an international normalized ratio known to be higher than 1.5 at the time of screening; abciximab bolus or infusion within the preceding 8 hours, or an eptifibatide or tirofiban bolus or infusion within the past 2 hours preceding randomization; or any other condition known to increase the risk of

bleeding.

- Severe concomitant condition or disease, such as cardiogenic shock at the time of randomization, ventricular arrhythmia refractory to treatment at the time of randomization, calculated creatinine clearance b 30 mL/min at screening, known significant liver disease (e.g., acute hepatitis, chronic active hepatitis, cirrhosis), or liver function test abnormalities (confirmed with repeat testing) which would require study drug discontinuation, i.e., aminoleucine transferase (ALT) more than 5 × the upper limit of the normal range (ULN) or ALT more than 3 × ULN plus total bilirubin more than 2 × ULN, prior ischemic stroke or transient ischemia attack, anemia (i.e., hemoglobin less than 10 g/dL＝ at screening, known clinical history of human immunodeficiency virus infection at screening, substance abuse (drug or alcohol) problem within the previous 6 months or any severe condition such as cancer that would limit life expectancy to less than 12 months.
- With an indication for long-term oral anticoagulation therapy such as atrial fibrillation, venous thromboembolism, or prior placement of a mechanical heart valve.
- With other contraindications for use of rivaroxaban and enoxaparin.
- Enrolled in another clinical study.

4.3 Informed consent was signed.

All hospitalized ACS patients who met the inclusion and exclusion criteria could be included in this study after signing the informed consent.

4.4 Randomization

Log into the Interactive Web Response System (website address http://222.247.43.186:8008/), enter information according to the prompts on the webpage, randomly obtain the group number, and give interventions according to the groups.

4.5 Treatment

4.5.1 Intervention method

(1) "Rivaxaban 2.5 mg BID" group: 2.5 mg twice a day. Daily oral medication time is fixed, 12 hours apart.

(2) "Rivaxaban 5.0 mg BID" group: 5.0 mg twice a day. Daily oral medication time is fixed, 12 hours apart.

(3) "Enoxaparin 1 mg/kg Q12H" group: twice a day, 1 mg/kg each time. The dose was strictly calculated according to the body weight before enrollment and was administered by nurses at a fixed time every day, with an interval of 12 hours.

4.5.2 Intervention duration

(1) The total duration does not exceed 8 days;

(2) Discontinue use more than 12 hours before revascularization, and no longer use after revascularization;

(3) No further application of the intervention after discharge.

4.5.3 Concomitant therapy

All patients received dual antiplatelet therapy with aspirin (100 mg once daily) and clopidogrel (75 mg once daily), and the other secondary prevention regimens for coronary heart disease were determined by the clinic.

4.5.4 Study Drug Interruption and Discontinuation

1) If the drug is stopped for more than 12 hours before PCI, for example, if PCI is performed at 8:00 in the morning tomorrow, only the second intervention will be stopped today;

2) If urgent revascularization is needed, the intraoperative heparin dosage should be adjusted according to ACT.

3) All intervention drugs should be used for no more than 8 days and should be stopped before discharge.

4.5.5 Emergency bleeding plan

1) Minor bleeding events: close monitoring without drug discontinuation.

2) Major bleeding or clinically related nonmajor bleeding events: discontinuation of the drug, recommended according to the "Chinese Expert Consensus on Enoxaparin Anticoagulation Therapy in Acute Coronary Syndrome", for shock patients with excessive blood loss and continuous active bleeding In addition to the infusion of crystalloids, colloids and fresh frozen plasma and some red blood cells, such as massive bleeding caused by overdose of anticoagulant drugs, slow intravenous protamine can neutralize the above symptoms (1 mg protamine can neutralize the above symptoms). The anticoagulant effect produced by 1 mg of this

product), protamine neutralizes the anti-Xa activity of this product by up to 60%. If necessary, hemostasis under the guidance of image intervention and compression should be performed according to the bleeding site.

4.6 Study endpoint

4.6.1 Study endpoint events

Study endpoint events included the primary endpoint and secondary endpoints.

The primary safety endpoint was bleeding events according to the ISTH definition during the 6-month follow-up period.

The primary efficacy endpoint was a composite of major adverse cardiac events (MACEs), including cardiac death, myocardial infarction, rerevascularization or stroke, during the 6-month follow-up period.

Secondary endpoint events included cardiac-related rehospitalization and all-cause death.

4.6.2 Endpoint Definitions for H-REPLACE study

4.6.2.1 Bleeding events defined by ISTH criteria

ISTH major bleeding: Hemoglobin drop of >2 g/dL, transfusion of >2 units packed red blood cells, symptomatic bleeding in a critical area, or fatal bleeding.

ISTH CRNM bleeding: Requires or prolongs hospitalization or results in laboratory testing, imaging, compression, a procedure, interruption of the study medication, or a change in concomitant therapies.

ISTH Minor bleeding: Overt bleeding that does not meet the criteria for CRNM or major bleeding.

4.6.2.2 The primary efficacy endpoint

The primary efficacy endpoint was a composite of major adverse cardiac events (MACEs), including cardiac death, myocardial infarction, rerevascularization or stroke, during the 6-month follow-up period.

Cardiac death: Attribution of death to a cardiovascular etiology includes acute myocardial infarction, sudden cardiac death, HF, stroke, cardiovascular procedure, cardiovascular hemorrhage, and other cardiovascular causes.

Myocardial infarction: The categorization and definitions of the types of myocardial infarction are derived from the “Fourth Universal Definition of Myocardial Infarction”, the “2014 AHA/ACC Guideline for the Management of Patients with Non-ST- Elevation Acute Coronary Syndromes”, and the “2015 ACC/AHA/SCAI Guideline for the Management of ST-Elevation Myocardial Infarction”.

Rerevascularization: coronary revascularization procedures needed to treat symptoms of myocardial ischemia or based solely on coronary anatomic characteristics during the first 6 months after initial myocardial revascularization.

Stroke: stroke is defined on the basis of the presence of acute infarction as demonstrated by imaging or based on the persistence of symptoms.

4.6.2.3 Secondary endpoints

Cardiac-related rehospitalization: cardiovascular readmission was defined as nonelective repeat hospitalization in all patients alive at discharge for one or more of the following: angina, MI, coronary artery bypass graft surgery (CABG), nonstaged/nonindex artery PCI, heart failure or stroke.

All-cause death was defined as death due to any cause

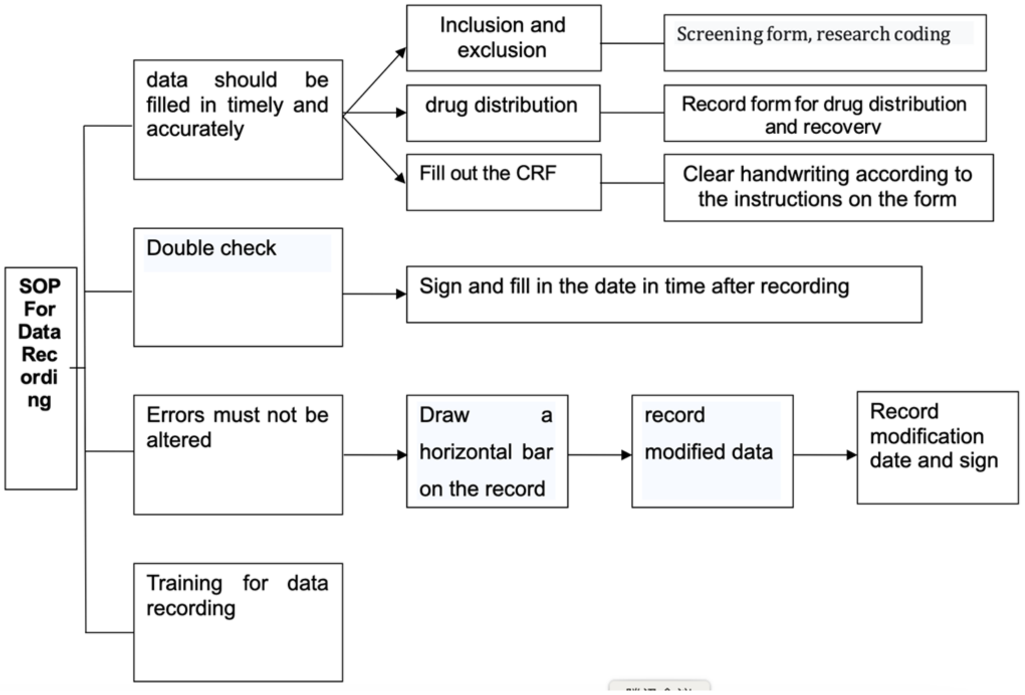

5. STUDY ASSESSMENTS AND PROCEDURES

5.1 Collecting data

To ensure data quality and integrity, all data requested to be collected should be documented in the CRF provided by the sponsor. Data will be entered into the CRF by the research center or designated party. During the study period, the CRF was completed promptly and accurately at each visit. The Principal Investigator or his designee should review and sign the CRF for accuracy. Completed CRFs should be

sent to the sponsor within 2 weeks of each visit.

5.1.1 After confirming that the subjects met the selection conditions and registering the baseline data, the specific contents included the following aspects:

(1) Demographic data, risk factors (hypertension, diabetes, etc.), past medical history (cardiovascular disease, CABG/PCI history, stroke/TIA, past bleeding history, etc.), previous treatment (including secondary prevention of coronary heart disease), antiarrhythmic drugs, other drugs, etc.), history of bad habits (smoking, drinking), and physical examination.

(2) Laboratory tests: liver function, kidney function, blood lipids, myocardial enzymes, coagulation function, blood routine, etc.

(3) Auxiliary examination: electrocardiogram (or dynamic electrocardiogram), cardiac echocardiography, etc.

(4) The total cost and details of inpatient treatment for ACS (detailed list of hospitalization costs).

5.1.2 Follow-up and data collection

After the subjects are enrolled in the study, they should be followed up 3 times according to the follow-up schedule and the medical routine of the selected center, that is, the first month of discharge and the third month of discharge. Completed by the follow-up team of the research center by telephone or in-office interview. Follow-up included clinically significant bleeding, cardiovascular/unknown cause death, myocardial infarction (including reinfarction), stroke (ischemic, hemorrhagic, and unexplained) and major ischemic events requiring repeat revascularization, any

rehospitalization for reasons, etc.

5.2 Standard Operating Procedures for Clinical Study Data Recording:

1) The clinical study data should be filled in the prescribed record book or predesigned form in a timely and accurate manner.
2) Fill in the screening and grouping form and the identification code table in time when screening the group.
3) Drug distribution was performed to fill in the drug distribution and recovery record form.
4) When filling in the original medical record, the data record should be clearly written, fill in the specified unit of measurement or fill in according to the instructions for filling in the form.
5) double check for review of the record by two reporters.
6) Regardless of the kind of original records, if any errors are found in the records, they shall not be altered. A horizontal bar shall be placed on the original records to ensure that the original records can be seen clearly, and then the modified data, modification date and signature shall be recorded.
7) Before the clinical start, provide training on the filling of all the original record forms provided, and provide instructions for filling in complicated ones.
8) Fill in the signature column of the recorder. After recording, you should sign

in time and fill in the date as needed.

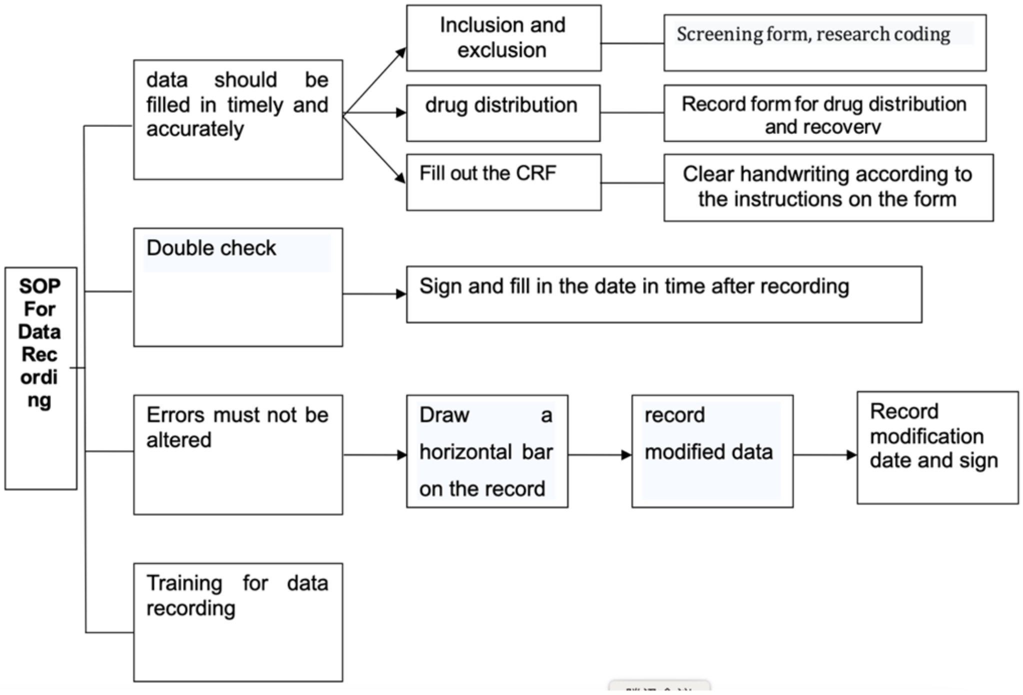

5.3 Trial management

5.3.1 Training of investigators, clinicians, recorders, enrolled patients:

1) Develop training programs to report research endpoint events for investigators, clinicians, recorders, and enrolled patients to avoid false positives and missed reports to the greatest extent possible.

2) Conduct simulation exercises for investigators, clinicians, recorders, and enrolled patients to report research endpoint events.

3) Special training on the identification, confirmation and reporting of research end-point events that occurred after discharge of enrolled patients.

4) Develop an SOP for reporting end-point events.

5) The report of the research end point event is finalized by the independent research end point event adjudication committee.

5.3.2 Recording, processing and reporting procedures:

1) Investigators, clinicians, and recorders who have undergone rigorous training and assessment have the responsibility and obligation to record and report research endpoint events in a timely and correct manner.

2) Suspicious events should be directly inquired about the events obtained by direct inquiries based on observations and questions similar to “Since the last inspection, how do you feel differently?” to ensure the objectivity of the inquiry results. The details of the study endpoint events were recorded as needed.

3) Investigators, clinicians and recorders who have undergone rigorous training and assessment shall report in strict accordance with the judgment standards in the research protocol.

4) After the 24-hour adverse event warning phone receives the suspicious event reported by the patient, it will be determined within 2 hours that one-to-one investigators will arrange for the tracking, identification and related data recording of the suspicious event and complete the report within 24 hours.

5.4 Patient Adherence Management

Since the intervention in this study was in-hospital treatment, compliance with

the study intervention was not involved. Therefore, the compliance of subjects with follow-up is the key to ensuring the quality of this study. Subject study compliance management should be initiated as early as possible and throughout the study.

5.4.1 Group information management

After signing the informed consent form, the contact information of the subjects and their families was kept as much as possible and recorded in the CRF form.

5.4.2 Communication Contact Management

After signing the informed consent form and obtaining the consent of the patients or their families, they were included in the WeChat patient follow-up management group. Patient follow-up reminders, suspicious event tracking, etc., can be carried out through WeChat, SMS and other communication forms.

5.4.3 Research information EDC system reminder

Set up the follow-up reminder function before the follow-up node to ensure that the subjects arrange the follow-up plan in time.

5.5 Subject completion/withdraw

5.5.1 Completion·

A subject will be considered to have completed the study if he or she has reached any endpoint before the end-of-study visit or completed the end-of-study visit.

5.5.2 Withdrawal from the Study

A subject will be withdrawn from the study for any of the following reasons:

- Lost to follow-up

• Withdrawal of consent

• Death

• Other (for example, where there is failure to meet inclusion/exclusion criteria, or when it is considered that the subject may be placed at an increased risk)

In case a subject is lost to follow-up, every possible effort will be made, including the possible use of locator agencies, to contact the subject and determine the endpoint status and reason for discontinuation as local law permits. The measures taken for follow-up must be documented. Subjects who withdraw consent for further study drug administration will be encouraged to remain in the study for endpoint event assessment. In addition, information on the final status will be obtained as frequently as necessary (if permitted by local law).

When a subject withdraws before completing the study, the reason for withdrawal is to be documented in the CRF and in the source document and will be contacted for survival status at the end of the study. Study drug assigned to the withdrawn subject may not be assigned to another subject. Subjects who withdraw will not be replaced.

5.6 Data management

An IDMC will be established to monitor the progress of the study and ensure that the safety of subjects enrolled in the study is not compromised. The IDMC will include, but is not limited to, a clinical chairman, physician(s) experienced in clinical studies, but not participating in this study, and at least 1 statistician. Details of the composition,

roles, responsibilities, and processes of the IDMC will be documented in its charter.

6. Statistical method

6.1 Noninferior margin

To determine the non-inferiority margin of the primary efficacy endpoint, we referred to the publication of NEJM. [12]

Regarding the primary safety endpoint (bleeding event), we had not found the published study with the same endpoint. We just estimated the potential margin according to the "fixed margin approach" recommended by FDA[13] based on the parameter regarding major bleeding events from a meta-analysis,[14] which reported LMWH was associated with a significantly increased risk of major bleeding (OR 2.26, 95% CI 1.63–3.14), therefore we could get the square root of the lower bound of the 95 percent confidence interval of 1.63, that is 1.28. Moreover, margin of (0.8, 1.25) is usually used in bioequivalence studies.[15] The non-inferiority margin of the primary safety endpoint was finally set at 1.24 in our trial with conservative clinical determination.

Both noninferior margins were determined before randomization of the first patient.

6.2 Sample Size Estimation

As a feasible pilot trial, approximately 2000 patients were expected to be enrolled during the study period.

6.3 Analysis Sets

ITT and PP analyses are planned for both safety and efficacy endpoints.

Intention-to-treat population (ITT): This analysis set included all randomized patients.

Per-protocol analysis (PP set): Refers to subjects who do not have serious protocol violations in terms of inclusion and exclusion criteria, treatment, and measurement of main indicators. The analysis was performed after the population withdrew from the study.

6.3 Statistical analysis

6.3.1 Baseline and demographic analysis

Depending on the type of data, a t test or analysis of variance will be used for measurement data, and the chi-square test will be used for enumeration data. Subgroup analyses of study endpoints will be performed based on baseline and demographic characteristics.

Age, sex, BMI, creatinine clearance, smoking, combined hypertension, diabetes, hyperlipidemia, previous myocardial infarction, previous PCI/CABG, previous stroke, type of ACS, revascularization received in current ACS, and drug therapy Wait.

6.3.2 Analysis of primary and secondary study endpoints

A Cox regression model was used to analyze the noninferiority of the direct event rates between the two low-dose rivaroxaban groups and the enoxaparin group and obtained HR and 95% confidence interval. The p value for inferiority was calculated based on the estimate, SE and cutoff value obtained from Cox regression,

and the formula was p value for noninferiority= PROBNORM[(estimate-LN(L))/SE)].

6.3.4 Subgroup analysis

According to population and baseline data, according to age (whether greater than 65 years old), BMI (whether greater than 25 kg/m2), creatinine clearance rate (whether less than 50 ml/min), smoking, past medical history (hypertension, diabetes, hyperlipidemia), subgroup analysis was performed for previous myocardial infarction, previous revascularization, stroke), and the type of current ACS, and an interaction p value less than 0.05 was considered to be different between subgroups.

7. Research Management

7.1 Research institutions

This project is a multicenter, prospective, randomized, controlled clinical study organized and implemented by the Second Xiangya Hospital of Central South University using a unified research protocol and CRF form. Each center entered the CRF form, which was compiled by the Second Xiangya Hospital of Central South University and was responsible for statistical analysis. After study termination/completion, subjects continued to receive routine care at each center.

Lead unit: The Second Xiangya Hospital of Central South University

Project leader: Professor Zhou Shenghua from the Second Xiangya Hospital of Central South University

7.2 Monitoring and Auditing

This study must strictly comply with the relevant regulations promulgated by the State Drug Administration of China, the Good Clinical Practice (GCP), and the Declaration of Helsinki (October 2000, Scotland).

Check the CRF form on a regular basis, and go to each center to monitor the original documents (select 10%-20% of the cases).

Raw files include printed, optical or electronic documents of raw data that can be used as raw files, such as hospital records, laboratory reports, instrument inventory records, radiographs, etc. Research centers and laboratories should keep original documents related to research. Copies of the original documents and printed copies of the original electronic documents should also be retained, signed and dated by the staff of the research center and marked as consistent with the original documents.

7.3 Management and preservation of files

Data collection method: A handwritten case report form was kept by each research center, and a CRF form was completed. Each center kept the electronic medical records of patients in its own unit, and the lead unit kept the electronic medical records of all centers. The electronic medical record is designed and maintained by the lead unit.

7.4 Consent procedure and change procedure of the experimental protocol

Each center can propose amendments to the test plan and report it to the lead unit.

After discussion, the changes were sent to each center.

8. Ethical requirements

Before the start of the study, the relevant documents will be submitted to the independent ethics committee (IEC) of the lead unit for review, and the implementation will begin after obtaining the approved documents. When the patients were selected, the research process was explained to the patients, the informed consent form was obtained, and the time was signed and recorded. Each center will protect the rights and interests of all subjects and keep personal data confidential.

9. Protocol Modifications

Modification 1: Follow-up time for primary endpoints

The follow-up time for primary endpoints was changed from 6 months to 12 months on March 2019 and changed back to 6 months on October 2020.

The follow-up time for the primary endpoints of the H-REPLACE study was set as 6 months in the NCT (https://clinicaltrials.gov/ct2/show/NCT03363035), which was registered on November 29, 2017. We submitted the rationale of the H-REPLACE study as a paper to a journal. We followed the reviewer's comments and agreed to change the follow-up time from 6 months to 12 months in the design paper. This change was made without authorization from the ethics committees of the Second Xiangya Hospital of Central South University. The ethics committees of the Second

Xiangya Hospital of Central South University discussed this issue and refused the change in October 2020. The initial objective of this study was to explore whether rivaroxaban can be a substitution for enoxaparin during the acute phase of ACS. The use of a 12-month time-window in the noninferiority assessment of safety and efficacy of an antithrombotic trial regimen given for the first 4 days only may increase the false-positive rate (alpha error) in the trial, as the addition of events that do not bear on the randomized treatment might increase the power of the trial. Therefore, we finally set the follow-up time for primary endpoints to 6 months with the approval of ethics committees. And we have also sent an application letter to the journal and publishers for the amendment of related contents in the published paper.

Modification 2: Sample size calculation

We found that the methods for sample size calculation were totally wrong during the period of the research execution. With the noninferiority margins mentioned above, it would have required sample sizes of 5655 of 18161 patients in each group. Furthermore, data monitoring committees found that the actual incidence of the primary endpoint was much lower than expected. Thus the principal investigator and steering committees finally decided to change the study to a feasibility trial with a sample size of approximately 2000 patients for the three arms.

10. References

[1] Amsterdam Ezra A, Wenger Nanette K, Brindis Ralph G, et al. 2014 AHA/ACC Guideline for the Management of Patients with Non-ST-Elevation Acute Coronary Syndromes: a report of the American College of Cardiology/American Heart Association Task Force on Practice Guidelines. J. Am. Coll. Cardiol, 2014, 64(24): e139-e228.

[2] Ibanez Borja, James Stefan, Agewall Stefan, et al. 2017 ESC Guidelines for the management of acute myocardial infarction in patients presenting with ST-segment elevation: The Task Force for the management of acute myocardial infarction in patients presenting with ST-segment elevation of the European Society of Cardiology (ESC). Eur. Heart J., 2017. doi:10.1093/eurheartj/ehx393

[2] Amsterdam Ezra A, Wenger Nanette K, Brindis Ralph G, et al. 2014 AHA/ACC Guideline for the Management of Patients with Non-ST-Elevation Acute Coronary Syndromes: a report of the American College of Cardiology/American Heart Association Task Force on Practice Guidelines. J. Am. Coll. Cardiol., 2014, 64(24): e139-e228.

[3] Heidbuchel Hein, Verhamme Peter, Alings Marco, et al. Updated European Heart Rhythm Association practical guide on the use of nonvitamin-K antagonist anticoagulants in patients with nonvalvular atrial fibrillation: Executive summary. Eur. Heart J., 2017, 38(27): 2137-2149.

[4] FDA: http://www.drugfuture.com/fda/drugview/020164

[5] FDA: http://www.drugfuture.com/fda/drugview/022406

[6] Kakkar Ajay K, Brenner Benjamin, Dahl Ola E, et al. Extended duration rivaroxaban versus short-term enoxaparin for the prevention of venous thromboembolism after total hip arthroplasty: a double-blind, randomized controlled trial. Lancet, 2008, 372(9632): 31-39.

[7] Prins Martin H, Bamber Luke, Cano Stefan J, et al. Patient-reported treatment satisfaction with oral rivaroxaban versus standard therapy in the treatment of pulmonary embolism; results from the EINSTEIN PE trial. Thromb. Res, 2015, 135(2): 281-288.

[8] Turpie Alexander G G, Lassen Michael R, Davidson Bruce L, et al. Rivaroxaban versus enoxaparin for thromboprophylaxis after total knee arthroplasty (RECORD4): a randomized trial. Lancet, 2009, 373(9676): 1673-1680.

[9] Beyer-Westendorf Jan, Lensing Anthonie W A, Arya Roopen, et al. Choosing wisely: The impact of patient selection on efficacy and safety outcomes in the EINSTEIN-DVT/PE and AMPLIFY trials. Thromb Res, 2017, 149: 29-37.

[10] Geller Bram J, Mega Jessica L, Morrow David A, et al. Autoantibodies to phosphorylcholine and cardiovascular outcomes in patients with acute coronary syndromes in the ATLAS ACS-TIMI 46 trial. J. Thromb. Thrombolysis, 2014, 37(3): 310-316.

[11] Spinler Sarah A, Inverso Stephanie M, Cohen Marc, et al. Safety and efficacy of unfractionated heparin versus enoxaparin in patients who are obese and patients with severe renal impairment: analysis from the ESSENCE and TIMI 11B studies. Am. Heart J, 2003, 146(1): 33-41.

[12] Fifth Organization to Assess Strategies in Acute Ischemic Syndromes Investigators, Yusuf Salim, Mehta Shamir R, et al. Comparison of fondaparinux and enoxaparin in acute coronary syndromes. N Engl J Med. 2006;354(14): 1464-1476.

[13] US Department of Health and Human Services, US Food and Drug Administration, Center for Biologics Evaluation and Research (CBER). Guidance for industry non-inferiority clinical trials. Published March 2010. Accessed April 8,2022.www.fda.gov/downloads/drugs/guidancecomplianceregulatoryinformation/guidances/ucm202140.pdf.

[14] Eikelboom JW, Anand SS, Malmberg K, Weitz JI, Ginsberg JS, Yusuf S. Unfractionated heparin and low-molecular-weight heparin in acute coronary syndrome without ST elevation: a meta-analysis. *Lancet*. 2000;355(9219):1936-1942.

[15] U.S. Department of Health and Human Services, Food and Drug Administration, Center for Drug Evaluation and Research (CDER). Guidance for industry: statistical Approaches to Establishing Bioequivalence. Published April 29, 2020. http://www.fda.gov/downloads/drugs/guidancecomplianceregulatoryinformation/guidances/ucm070244.pdf.

Statistical Analysis Plan

Study Code	<< NCT03363035>>
Edition Number	<< 4.0>>
Date	<<20/October/2020>>

Safety and efficacy of low-dose rivaroxaban in the acute phase of acute coronary syndrome: an open-label, randomized, noninferiority comparison (H-REPLACE)

TABLE OF CONTENTS

TITLE PAGE 1
TABLE OF CONTENTS 2
AMENDMENT HISTORY 4
1 INTRODUCTION 5
2 STUDY OBJECTIVES 6
3 STUDY DESIGN 7
4 GENERAL STATISTICAL CONSIDERATIONS 8
4.1 General Principles 8
4.2 Handling of Noncompliance to Study Treatment or Follow up 9
4.3 Handling of Missing Data 10
4.4 Data Monitoring 11
4.5 Determination of sample size 12
4.6 Determination of Noninferiority margins 12
5 ANALYSIS SETS 13
5.1 Assignment of analysis sets 13
5.1.1 Intention-to-treat analysis set (ITT) 13
5.1.2 Per-protocol analysis set (PP) 13
5.1.3 Safety analysis set (SAF) 13
6 STATISTICAL METHODOLOGY 14
6.1 Population characteristics 14
6.1.1 Demographics 14
6.1.2 Medical history 15
6.1.3 Protocol Deviations 15
6.1.4 Prior and concomitant medications 16
6.2 Efficacy 16
6.2.1 Primary efficacy variable 16

6.2.2 Primary safety variable 16
6.2.3 Secondary efficacy variable 17
6.2.4 Subgroup variables 17
6.2.5 Sequential analyses 18
6.2.6 Analysis of the primary safety variable 19
6.2.7 Analysis of the primary efficacy variable 19
6.2.8 Analysis of the secondary efficacy variable 20
6.2.9 Subgroup Analyses 20
6.2.10 Sensitivity analyses 20
7 DOCUMENT HISTORY AND CHANGES IN THE PLANNED STATISTICAL ANALYSIS 21
7.1 Overview Changes to SAP 21
8 REFERENCES 23

AMENDMENT HISTORY

Category*: Change refers to	Date	Description of change
<<Followup time>>	<<3/March/2019>>	<< The follow-up time for primary endpoints were changed from 6 months to 12 months on March 2019 and changed back to 6 months on October 2020 due to clinical consideration.>>
<<Sample size calculation and study type>>	<<10/October/2020>>	<< Methods for sample size calculation was wrong and the study type was changed to be feasibility trial with a sample size around 2000 patients>>

1 INTRODUCTION

Cardiovascular disease death accounts for the first place in the total deaths of urban and rural residents in China, with 45% in rural areas and 43% in urban areas; coronary heart disease is an important cause of death, and acute coronary syndrome (ACS) is the main cause of coronary heart disease. Types of. ACS is a group of clinical syndromes caused by acute myocardial ischemia, including unstable angina (UA), ST-segment elevation myocardial infarction (STEMI), and non-ST-segment elevation myocardial infarction (NSTEMI). ACS is characterized by acute onset, rapid changes in condition, and poor prognosis. The hospitalized and long-term mortality rates are 6% and 12%, respectively [1], which seriously endangers social and public health.

Regardless of the subtype of ACS, thrombosis and/or thromboembolism are the predominant pathophysiological mechanisms. Therefore, anticoagulation drugs play an extremely important role in the treatment of ACS. Platelet activation and coagulation system activation are two crucial links in the process of thrombosis and/or thromboembolism; the two are closely linked in the body, and thrombin generated after coagulation system activation is a powerful platelet activating factor. Activation of platelets in turn promotes the coagulation process. Antithrombotic therapy should target both the coagulation system (anticoagulation therapy) and platelets (antiplatelet therapy). DAPT therapy is the cornerstone of the treatment of ACS patients and has received enough attention in clinical practice, while anticoagulation therapy is often neglected. Based on evidence from a large number of high-quality studies, guidelines[2,3] clearly recommend that short-term (less than 8 days) combined use of anticoagulant and antiplatelet drugs for ACS can reduce the incidence of thrombotic events

and improve prognosis in patients with ACS. Enoxaparin is a low molecular weight heparin with the strongest anticoagulant effect. The short-term combined use of enoxaparin anticoagulation and aspirin + P2Y12 receptor antagonist dual antiplatelet (DAPT) is a class IA recommendation for patients with UA, NSTEMI, and STEMI during conservative drug treatment or waiting for PCI.

The peak time and half-life of rivaroxaban are superior or non-inferior to enoxaparin[4-6], and rivaroxaban is an oral drug and does not require subcutaneous injection; the dose is fixed and does not need to be adjusted by body weight. Can increase compliance. Clinical studies related to anticoagulation in patients with pulmonary embolism/deep vein thrombosis and hip/knee replacement [7-9] have confirmed that the safety and efficacy of rivaroxaban are superior or non-inferior to enoxaparin.

The ATLAS ACS TIMI 46 study [10] showed that the incidence of clinically significant bleeding (approximately 1.5%-2.5%) in 2.5 mg BID and 5 mg BID in the short term (8 days) with rivaroxaban combined with DAPT was much lower than that of enoxaparin. The incidence of bleeding in combination with DAPT is 3% to 9% [11].

In conclusion, we hypothesized that the safety and efficacy of short-term anticoagulation with rivaroxaban during hospitalization in ACS patients treated with DAPT is non-inferior to enoxaparin.

2 STUDY OBJECTIVES

To compare the safety and efficacy of rivaroxaban versus enoxaparin in patients with ACS who missed the primary reperfusion therapy window and before selective revascularization.

2.1 Primary objective

1) Is the bleeding risk (ISTH bleeding) of short-term in-hospital low-dose rivaroxaban in ACS patients non-inferior to low-molecular-weight heparin?

2) Is the risk of cardiovascular events (cardiac death, myocardial infarction (including reinfarction), stroke (ischemic, hemorrhagic and unexplained) and the need for repeat blood in ACS patients with short-term low-dose rivaroxaban in hospital noninferior to low molecular weight heparin?

2.2 Secondary objective

1) Can short-term in-hospital low-dose rivaroxaban in ACS patients reduce psychogenic hospitalization compared with low-molecular-weight heparin?

2) Can short-term in-hospital application of low-dose rivaroxaban in ACS patients reduce all-cause mortality compared with low-molecular-weight heparin?

3 STUDY DESIGN

This study is a prospective, multicenter, open-label, randomized, active-controlled, noninferiority feasibility study.

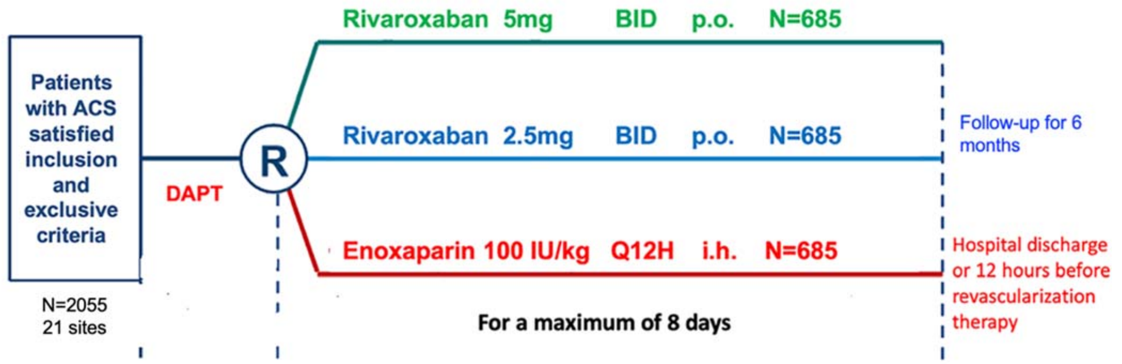

This prospective, randomized, open-label, active-controlled, multicenter study is designed to compare the safety and efficacy of rivaroxaban versus enoxaparin in patients with ACS who missed the primary reperfusion therapy window and before selective revascularization. Participants receiving background treatment of aspirin plus clopidogrel or ticagrelor will be randomly assigned to either oral rivaroxaban 2.5 mg twice daily or rivaroxaban 5 mg twice daily or subcutaneous enoxaparin 1 mg/kg twice daily until hospital discharge for a maximum of 8 days or 12 h before revascularization therapy. The primary safety endpoint is the International Society on Thrombosis and Hemostasis definition of bleeding events [minor, clinically relevant nonmajor and major bleeding]. The primary efficacy endpoint is a composite of major adverse cardiac events (MACEs), including cardiac death, myocardial infarction, rerevascularization or stroke, and major bleeding events. Secondary endpoints include cardiac-related rehospitalization and all-cause death. Patients will be followed for 6 months after randomization.

4 GENERAL STATISTICAL CONSIDERATIONS

4.1 General Principles

The variables will be analyzed using descriptive statistics. As appropriate, metric data will be

calculated to be the number of available data and missing data, the mean, the standard deviation, the minimum, the quartiles, the median, and the maximum. Frequency tables will be generated for categorical data. The statistical analysis will be performed using SAS version 9.2 or higher (SAS Institute Inc., Cary, NC, USA).

4.2 Handling of Noncompliance to Study Treatment or Follow up

Patients who discontinue treatment or study follow-up at an excessive rate may make the trial unintelligible. Data on outcomes and vital status will be collected throughout the study, even if patients stop taking study medications. Hence, all randomized patients' clinical data will be collected at the trial close-out as far as possible.

Subjects who sign an informed consent form and, for any reason (e.g., failing to meet inclusion and exclusion criteria), terminate the study before being randomized are referred to as a "screening failure".

For any reason, a randomized subject who permanently discontinued the study treatment before their End of Treatment Visit was considered to have permanently discontinued the study medication (including subjects who were randomized but never started taking any study medication). Study medication will be permanently discontinued based on the reason for discontinuation.

If subjects permanently discontinued study medication, different options of follow-up were discussed to collect outcome events and vital status. It can include regular study visits, regular telephone contacts with the patient or the general practitioner, or contact at the end of the study. If patients do not agree to attend regular study visits, the investigator will encourage

them to attend at least one final assessment visit to the greatest extent possible as outlined for the End of treatment Visit.

All randomized subjects will be encouraged to remain on study treatment and under observation for the duration of the study. Informed consent cannot be withdrawn by discontinuing study treatment. In cases where subjects indicate they do not want to “continue”, investigators must determine whether this refers to discontinuation of study treatment, unwillingness to attend follow-up visits, unwillingness to have telephone contact, unwillingness to have any contact with study personnel, or unwillingness to allow contact with a third party (e.g., family member, doctor). To determine the survival status of all subjects at the end of the study, every effort will be made to follow up on them. Only a very small number of individuals are anticipated to have incomplete follow-up (in any manner) during this trial.

A subject will be declared to have incomplete follow-up or to be lost to follow-up (i.e., to be completely noncompliant to follow-up) if, despite all possible efforts, all investigators and dedicated site staff are not able to contact the subject or a third party (e.g., family member, doctor). As local law allows, every effort will be made to contact the subject or a third party and to determine the endpoint, survival status, and reason for discontinuation. The subject will not be classified as lost to follow-up if the database documents that the subject is alive at the end of the study.

4.3 Handling of Missing Data

In the subject data listing, all missing or partial data will be listed as they appear on the CRF, including the best estimate date of site investigators (see below) collected in the clinical data.

Missing or incomplete event dates

Site investigators are asked to provide their best estimate of the event date when an event date is unknown. Although the precise date of an occurrence is unknown, the investigator frequently has knowledge of details that would indicate the date's approximation, such as the first week of a month, in the fall of a year, or the middle of a particular year, or at least the date when the subject was last seen or contacted. It is likely that the estimated date recorded by the computer program will be closer to the true date than the date produced by an uninformed computer program if this information is incorporated into it. The estimated date should be the middle date within the period when the event is known to have taken place. A date in the middle of a month should be used as an estimate if the event occurred in the first week of the month. The middle date in the fall is the appropriate estimate if it occurred in the fall. I The date in the middle of the plausible time period should be given if no information is known about the subject prior to the event (start date of plausible time period) and the date of contact when information about the event was known (end date of plausible time period). It has been used in many studies and is recommended by Dubois for date estimation [12].

To be conservative, if date/time information is not sufficient to determine whether an event occurred before or after randomization, it is considered an outcome. The event start date will be imputed no earlier than the randomization date.

4.4 Data Monitoring

To assess the efficacy and safety of the study, the IDMC will review unblinded event rates. All (unblinded) statistical analyses for the IDMC will be performed by the ISAC (Independent Statistical Analysis Centre).

Overall blinded event rates will be reviewed by the steering committee to ensure that they meet protocol projections. The trial design may be changed if overall event rates are lower than expected, including an increase in sample size or an extension of study duration without prior knowledge of any treatment effect.

4.5 Determination of sample size

4.6 Determination of Noninferiority margins

To determine the non-inferiority margin of the primary efficacy endpoint, we referred to the publication of NEJM[14] (Comparison of fondaparinux and enoxaparin in acute coronary syndromes, the margin was 1.185 in this paper).

Regarding the primary safety endpoint (bleeding event), we did not find a published study with the same endpoint. We estimated the potential margin according to the "fixed margin approach" [15] recommended by the FDA based on the parameter regarding major bleeding events from a meta-analysis,[13] which reported that LMWH was associated with a significantly increased risk of major bleeding (OR 2.26, 95% CI 1.63–3.14); therefore, we obtained the square root of the lower bound of the 95% confidence interval of 1.63, that is, 1.28. Moreover, a margin of (0.8, 1.25) is usually used in bioequivalence studies. [16] The non-inferiority margin of the primary safety endpoint was finally set at 1.24 in our trial with conservative clinical determination.

5 ANALYSIS SETS

As a pilot feasible trial, around 2000 patients were expected to be enrolled during the study period.

5.1 Assignment of analysis sets

All subjects who were randomized in the H-REPLACE study were eligible for assignment to the analysis sets.

5.1.1 Intention-to-treat analysis set (ITT)

All participants who were randomly assigned will be included in the intention-to-treat analysis set, also known as the full analysis set in the International Conference on Harmonization (ICH) E9 standard.

5.1.2 Per-protocol analysis set (PP)

PP set Refers to subjects who do not have serious protocol violations in terms of inclusion and exclusion criteria, treatment, and measurement of main indicators. The analysis was performed after the population withdrew from the study.

5.1.3 Safety analysis set (SAF)

All randomized subjects who received at least one dose of study medication will be included in the safety analysis set.

6 STATISTICAL METHODOLOGY

6.1 Population characteristics

6.1.1 Demographics

In the ITT and SAF, baseline characteristics and demographics will be summarized by treatment group and overall. Metric variables will be presented in summary statistics. Frequency tables will be presented for categorical variables.

An individual's demographic information includes their age, gender, race, ethnicity, body height, weight, and body mass index (BMI). Age, body weight, and BMI will each be given as continuous variables and categorized into the following categories:

- Age: <65; ≥75 years
- BMI: < 25; ≥ 25kg/m^2

The following additional baseline characteristics will be analysed:

- creatinine clearance: <50; ≥50 mL/min/1.73m^2
- Hypertension: yes or no
- Diabetes: yes or no
- Dyslipidemia: yes or no
- Heart failure: yes or no
- Previous myocardial infarction

- Previous PCI or CABG
- Previous stroke
- Tobacco use: never, former, current
- Index diagnosis: STEMI, NSTEMI or unstable angina
- Medication use: beta-blocker, ACE inhibitor or ARB, statin, calcium-channel block
- Reasons for missing opportunity for direct reperfusion: Patient delay, Financial issues, Misdiagnosis, Others

6.1.2 Medical history

We will use frequency tables to evaluate the medical history data, showing how many subjects have medical history findings (i.e., listed conditions of previous diagnoses, diseases, or surgeries based on the CRF) that started before signing the informed consent and that are considered relevant to the study.

6.1.3 Protocol Deviations

The frequency tables show the number of subjects with major protocol deviations overall, grouped by type of deviation. The analysis will be based on ITT data. Types of major protocol deviations include the following:

- Significant inclusion criteria not fulfilled
- Significant exclusion criteria fulfilled
- Failure to obtain informed consent before initiation of study procedures

- Prohibited medication use
- Failure to report SAE
- Failure to report outcome event
- Other safety

6.1.4 Prior and concomitant medications

Frequency tables by type of medication will be provided for prior medications prior to the index events (for antiplatelets and anticoagulants) and prior to randomization (for all) and separately for concomitant medication continued after randomization, for visits 1, 3, 6 months, at EOT. Analyses will be by treatment group and overall based on ITT.

6.2 Efficacy

6.2.1 Primary efficacy variable

The primary efficacy variable is the time from randomization to the first occurrence of any of the components of the composite outcome, including

- Cardiac death
- Myocardial infarction
- Rerevascularization
- Stroke

6.2.2 Primary safety variable

The primary efficacy variable is the time from randomization to the first occurrence of ISTH bleeding events:

Event Classification	Definition
ISTH major bleeding	Hemoglobin drop of >2 g/dL, transfusion of >2 units packed red blood cells, symptomatic bleed in a critical area, or fatal bleeding
ISTH CRNM bleeding	Requires or prolongs hospitalization or results in laboratory testing, imaging, compression, a procedure, interruption of the study medication, or a change in concomitant therapies
ISTH Minor	Overt bleeding that does not meet criteria for CRNM or Major bleeding

6.2.3 Secondary efficacy variable

The secondary efficacy variables of this study are the time from randomization to first occurrence of the following:

- Cardiac-related rehospitalization
- All-cause death

6.2.4 Subgroup variables

For the comparison of primary efficacy and safety outcomes, the following subgroup analyses will be conducted based on baseline demographics:

- Age: <65; 60-75; >75 age (for publication: <65; ≥65 years) additionally <60; >=60 years
- Sex: male; female
- BMI: < 25; ≥ 25 to < 30; ≥ 30kg/m^2 (for publication: < 25; ≥ 25kg/m^2)
- Weight: <70; 70-90; >90 kg (for publication: <50; 50-100; >100 kg)
- creatinine clearance: <50; 50-80; >80 mL/min (for publication: <50; ≥50 mL/min)
- Smoking: ever, former, current (for publication: yes or no)
- Hypertension: yes or no
- Dyslipidemia: yes or no
- Diabetes: yes or no
- Heart failure: yes or no
- Previous myocardial infarction
- Previous PCI or CABG
- Previous stroke
- Tobacco use: never, former, current
- Index diagnosis: STEMI, NSTEMI or unstable angina

The Steering Committee may propose a limited number of additional subgroup analyses.

6.2.5 Sequential analyses

Sequential noninferiority analyses were used for the primary endpoints. The coprimary endpoints were analyzed using a fixed-sequence testing procedure in order of (i) safety endpoint between the rivaroxaban-2.5 mg and enoxaparin groups, (ii) safety endpoint between the rivaroxaban-5 mg and enoxaparin groups, (iii) efficacy endpoint between the rivaroxaban-

5 mg and enoxaparin groups, and (iv) efficacy endpoint between the rivaroxaban-2.5 mg and enoxaparin groups. The noninferiority analysis was to be followed by a superiority analysis if noninferiority analysis was concluded.

6.2.6 Analysis of the primary safety variable

The primary analysis will include events adjudicated by the ICAC and will be based on the ITT analysis set using the ITT data scope.

To evaluate whether low-dose rivaroxaban is noninferior to enoxaparin in prolonging the time to a primary safety (PS) outcome event in patients with ACS, the following null hypothesis (H0) will be tested at the significance level of 0.025:

H_0: PS1/PS2 ≤ 1.24 versus

H_1: PS1/PS2 ＞ 1.24

where PE1 is the incidence of PS events for the low-dose rivaroxaban group and PS2 is the incidence of PE outcome for the enoxaparin group. The ratio of 1.24 is the noninferior margin determined by the methods mentioned in section 4.6.

6.2.7 Analysis of the primary efficacy variable

ICAC events will be included in the primary analysis based on the ITT data scope and the ITT analysis set. To evaluate whether low-dose rivaroxaban is noninferior to enoxaparin in prolonging the time to a primary efficacy (PE) outcome event in patients with ACS, the following null hypothesis (H0) will be tested at the significance level of 0.025:

H_0: PE1/PE2 $\leq$ 1.185 versus

H_1: PE1/PE2 $>$ 1.185

where PE1 is the incidence of PE outcome for the low-dose rivaroxaban group and PE2 is the incidence of PE outcome for the enoxaparin group. The ratio of 1.185 is the noninferior margin determined by the methods mentioned in section 4.6.

6.2.8 Analysis of the secondary efficacy variable

The first secondary objective (to compare all-cause death and cardiac-related rehospitalization between the two groups) will be assessed via the following superiority hypotheses:

H0: SE1/SE2 $\leq$ 1.0 versus

H1: SE1/SE2 $>$ 1.0

where SE1 is the incidence of SE outcome for the low-dose rivaroxaban group and SE2 is the incidence of SE outcome for the enoxaparin group.

6.2.9 Subgroup Analyses

To determine whether the intervention effect is consistent across various subgroups, the estimate of the between-group treatment effect (with a nominal 95% CI) will be summarized for the primary endpoint.

6.2.10 Sensitivity analyses

To support the primary study results and to assess the robustness of the primary analysis, several sensitivity analyses will be conducted. For the sensitivity analyses, the per-protocol

population was used.

7 DOCUMENT HISTORY AND CHANGES IN THE PLANNED STATISTICAL ANALYSIS

- Approval of the SAP dated 22 November 2017
- Amendment 1 of the SAP dated 6 March 2019
- Amendment 2 of the SAP dated 10 October 2020

7.1 Overview Changes to SAP

The changes described in this section are editorial, administrative, and typographical corrections that do not affect the overall integrated SAP.

The following changes are introduced in SAP Version 2.0.

Modification 1: Follow-up time for primary endpoints

The follow-up time for primary endpoints was changed from 6 months to 12 months on March 2019 and changed back to 6 months on October 2020.

The follow-up time for the primary endpoints of the H-REPLACE study was set as 6 months in the NCT (https://clinicaltrials.gov/ct2/show/NCT03363035), which was registered on November 29, 2017. We submitted the rationale of the H-REPLACE study as a paper to a journal. We followed the reviewer's comments and agreed to change the follow-up time from

6 months to 12 months in the design paper. This change was made without authorization from the ethics committees of the Second Xiangya Hospital of Central South University. The ethics committees of the Second Xiangya Hospital of Central South University discussed this issue and refused the change in October 2020. The initial objective of this study was to explore whether rivaroxaban can be a substitution for enoxaparin during the acute phase of ACS. The use of a 12-month time-window in the noninferiority assessment of safety and efficacy of an antithrombotic trial regimen given for the first 4 days only may increase the false-positive rate (alpha error) in the trial, as the addition of events that do not bear on the randomized treatment might increase the power of the trial. Therefore, we finally set the follow-up time for primary endpoints to 6 months with the approval of ethics committees. And we have also sent application letter to the journal and publishers for amendment of related contents in the published paper.

Modification 2: Sample size calculation

Initial wrong methods:

The sample size was calculated on the website http://powerandsamplesize.com/Calculators/Compare-2-Proportions/2-Sample-Non-Inferiority-or-Superioritypower.

According to the data from a meta-analysis[13], the event rate was 1.5% for 2.5 mg rivaroxaban, 1.8% for 5 mg rivaroxaban, and 4.3% for enoxaparin, the beta value was set at 0.8, the alpha value was set at 0.025, the delta value was 1/10 (0.0043), and the obtained sample size was 780 for 2.5 mg rivaroxaban and 1076 for 5 mg rivaroxaban.

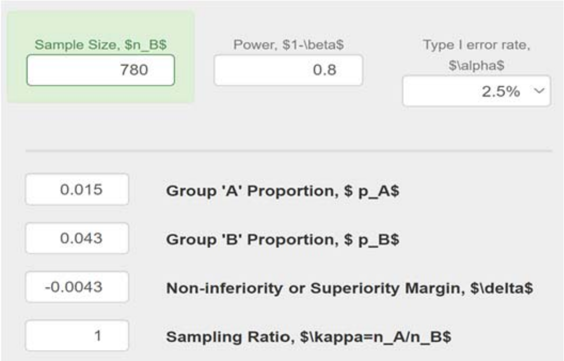

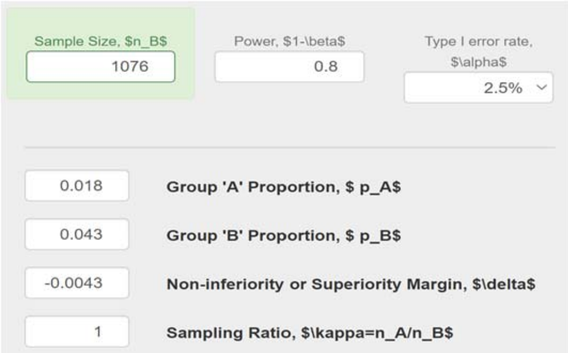

N=1076 was taken plus an assumed 5% loss-to-follow-up rate, N=1130 for each was assumed, so 3390 was submitted to the clinicaltrials website.

We found that the methods for sample size calculation was totally wrong as we calculated the sample assuming the events rate of the two group was not equal during the period of the research execution. With the noninferiority margins mentioned above, it would have required sample sizes of 5655 and of 18161 patients in each group. Furthermore, data monitoring committees found that the actual incidence of the primary endpoint was much lower than expected. Thus the principal investigator and steering committees finally decided to change the study to a feasibility trial with a sample size of approximately 2000 patients for the three arms.

8 REFERENCES

[1] Amsterdam Ezra A, Wenger Nanette K, Brindis Ralph G, et al. 2014 AHA/ACC Guideline for the Management of Patients with Non-ST-Elevation Acute Coronary Syndromes: a report

of the American College of Cardiology/American Heart Association Task Force on Practice Guidelines. J. Am. Coll. Cardiol, 2014, 64(24): e139-e228.

[2] Ibanez Borja, James Stefan, Agewall Stefan, et al. 2017 ESC Guidelines for the management of acute myocardial infarction in patients presenting with ST-segment elevation: The Task Force for the management of acute myocardial infarction in patients presenting with ST-segment elevation of the European Society of Cardiology (ESC). Eur. Heart J., 2017. doi:10.1093/eurheartj/ehx393

[2] Amsterdam Ezra A, Wenger Nanette K, Brindis Ralph G, et al. 2014 AHA/ACC Guideline for the Management of Patients with Non-ST-Elevation Acute Coronary Syndromes: a report of the American College of Cardiology/American Heart Association Task Force on Practice Guidelines. J. Am. Coll. Cardiol., 2014, 64(24): e139-e228.

[3] Heidbuchel Hein, Verhamme Peter, Alings Marco, et al. Updated European Heart Rhythm Association practical guide on the use of non-vitamin-K antagonist anticoagulants in patients with non-valvular atrial fibrillation: Executive summary. Eur. Heart J., 2017, 38(27): 2137-2149.

[4] FDA: http://www.drugfuture.com/fda/drugview/020164

[5] FDA: http://www.drugfuture.com/fda/drugview/022406

[6] Kakkar Ajay K, Brenner Benjamin, Dahl Ola E, et al. Extended duration rivaroxaban versus short-term enoxaparin for the prevention of venous thromboembolism after total hip arthroplasty: a double-blind, randomised controlled trial. Lancet, 2008, 372(9632): 31-39.

[7] Prins Martin H, Bamber Luke, Cano Stefan J, et al. Patient-reported treatment satisfaction with oral rivaroxaban versus standard therapy in the treatment of pulmonary embolism; results from the EINSTEIN PE trial. Thromb. Res, 2015, 135(2): 281-288.

[8] Turpie Alexander G G, Lassen Michael R, Davidson Bruce L, et al. Rivaroxaban versus enoxaparin for thromboprophylaxis after total knee arthroplasty (RECORD4): a randomised trial. Lancet, 2009, 373(9676): 1673-1680.

[9] Beyer-Westendorf Jan, Lensing Anthonie W A, Arya Roopen, et al. Choosing wisely: The impact of patient selection on efficacy and safety outcomes in the EINSTEIN-DVT/PE and AMPLIFY trials. Thromb Res, 2017, 149: 29-37.

[10] Geller Bram J, Mega Jessica L, Morrow David A, et al. Autoantibodies to phosphorylcholine and cardiovascular outcomes in patients with acute coronary syndromes in the ATLAS ACS-TIMI 46 trial. J. Thromb. Thrombolysis, 2014, 37(3): 310-316.

[11] Spinler Sarah A, Inverso Stephanie M, Cohen Marc, et al. Safety and efficacy of unfractionated heparin versus enoxaparin in patients who are obese and patients with severe renal impairment: analysis from the ESSENCE and TIMI 11B studies. Am. Heart J, 2003, 146(1): 33-41.

[12] Dubois M F, Hbert R. Imputation of Missing Dates of Death or Institutionalization for Time-to-Event Analyses in the Canadian Study of Health and Aging. International Psychogeriatrics, 2001, 13 supp 1(S1):91-97.

[13] Eikelboom JW, Anand SS, Malmberg K, Weitz JI, Ginsberg JS, Yusuf S. Unfractionated heparin and low-molecular-weight heparin in acute coronary syndrome without ST elevation:

a meta-analysis. *Lancet*. 2000;355(9219):1936-1942.

[14] Fifth Organization to Assess Strategies in Acute Ischemic Syndromes Investigators, Yusuf Salim, Mehta Shamir R, et al. Comparison of fondaparinux and enoxaparin in acute coronary syndromes. N Engl J Med. 2006;354(14): 1464-1476.

[15] US Department of Health and Human Services, US Food and Drug Administration, Center for Biologics Evaluation and Research (CBER). Guidance for industry non-inferiority clinical trials. Published March 2010. Accessed April 8,2022.www.fda.gov/downloads/drugs/guidancecomplianceregulatoryinformation/guidances/ucm202140.pdf.

[16] U.S. Department of Health and Human Services, Food and Drug Administration, Center for Drug Evaluation and Research (CDER). Guidance for industry: statistical Approaches to Establishing Bioequivalence. Published April 29, 2020. Accessed April 8, 2022. http://www.fda.gov/downloads/drugs/guidancecomplianceregulatoryinformation/guidances/ucm070244.pdf.
