# Supplementary material for: Effect of Rivaroxaban vs Enoxaparin on Major Cardiac Adverse Events and Bleeding Risk in the Acute Phase of Acute Coronary Syndrome: The H-REPLACE Randomized Equivalence and Noninferiority Trial
Source: JAMA Netw Open. 2023 Feb 10;6(2):e2255709. doi: 10.1001/jamanetworkopen.2022.55709 (PMC9918885; doi:10.1001/jamanetworkopen.2022.55709)
Supplement: Supplement 2. — eMethods. Protocol Amendment eAppendix 1. Inclusion and Exclusion Criteria eAppendix 2. Periprocedural Antithrombotic Management eAppendix 3. End Point Definitions eAppendix 4. Standard Operation Procedure for Adjudication Process eAppendix 5. Determination of Noninferior Margin eTable 1. Definitions of ISTH Bleeding Events eTable 2. Baseline Characteristics (ITT Analysis) eTable 3. Primary and Secondary End Points (ITT Analysis) eFigure 1. Risk of Primary Safety End Point Based on Major Subgroup eFigure 2. Risk of Primary Efficacy End Point Based on Major Subgroup eReferences. [file jamanetwopen-e2255709-s002.pdf]

Supplemental Online Content

Zhou S, Xiao Y, Zhou C, et al; H-REPLACE Investigators. Effect of rivaroxaban vs enoxaparin on major cardiac adverse events and bleeding risk in the acute phase of acute coronary syndrome: the H-REPLACE randomized equivalence and noninferiority trial. *JAMA Netw Open.* 2023;6(2):e2255709. doi:10.1001/jamanetworkopen.2022.55709

eMethods. Protocol Amendment
eAppendix 1. Inclusion and Exclusion Criteria
eAppendix 2. Periprocedural Antithrombotic Management
eAppendix 3. End Point Definitions
eAppendix 4. Standard Operation Procedure for Adjudication Process
eAppendix 5. Determination of Noninferior Margin
eTable 1. Definitions of ISTH Bleeding Events
eTable 2. Baseline Characteristics (ITT Analysis)
eTable 3. Primary and Secondary End Points (ITT Analysis)
eFigure 1. Risk of Primary Safety End Point Based on Major Subgroup
eFigure 2. Risk of Primary Efficacy End Point Based on Major Subgroup
eReferences.

This supplemental material has been provided by the authors to give readers additional information about their work.

© 2023 Zhou S et al. *JAMA Network Open.*

eMethods. Protocol Amendment

Details of the protocol [1] and amendments are described below. All the protocol amendments stated above were approved by the ethics committees of the Second Xiangya Hospital of Central South University.

Two major modifications were made during the research.

Modification 1: Follow-up time for primary endpoints

The follow-up time for primary endpoints was changed from 6 months to 12 months on March 2019 and changed back to 6 months on October 2020.

The follow-up time for the primary endpoints of the H-REPLACE study was set as 6 months in the NCT (https://clinicaltrials.gov/ct2/show/NCT03363035), which was registered on November 29, 2017. We submitted the rationale of the H-REPLACE study for publication consideration. One reviewer suggested us to change the follow up time from 6 months to 12 months to increase the number of patients reaching outcomes, we followed the reviewer’s comments and agreed to change the follow-up time from 6 months to 12 months in the design paper. This change was made without authorization from the ethics committees of the Second Xiangya Hospital of Central South University. The ethics committees of the Second Xiangya Hospital of Central South University discussed this issue and refused the change in October 2020. The consideration was that study patients were already enrolled on March 2019, and the extension to 12-month follow up might increase the false-positive rate (alpha error) in the trial, as the addition of events might be unrelated to the study drugs, which was applied at the acute phase of ACS for about 4 days.

© 2023 Zhou S et al. *JAMA Network Open.*

Therefore, the follow-up time for primary endpoints was set back to 6 months with the approval of ethics committees. And we have also sent an application letter to the journal and publishers for the amendment of related contents in the published paper.

Modification 2: Sample size calculation

We realized that the methods for sample size calculation were not correct during the period of the research execution. With the noninferiority margins mentioned above, a sample sizes of 5655 patients would be required in each group (total 18161). Furthermore, data monitoring committees found that the actual incidence of the primary endpoint was much lower than expected. Thus, the principal investigator and steering committees finally decided to change the study to a feasibility trial with a sample size of approximately 2000 patients for the three arms with the approval of ethics committees.

As stated above, the sample size was not re-estimated as the principal investigator and steering committees finally decided to change the study to a feasibility trial.

© 2023 Zhou S et al. *JAMA Network Open.*

eAppendix 1. Inclusion and Exclusion Criteria

**Inclusion criteria**
● Male or female aged more than 18 years
● Diagnosed with ACS (STEMI, NSTEMI, unstable angina) who missed primary reperfusion window and before selective revascularization
● With an indication for short-term combination use of DAPT and enoxaparin.
**Exclusion criteria**
● Already received thrombolytic therapy or revascularization or needing revascularization therapy in 12 hours.
● With platelet glycoprotein IIb/IIIa receptor antagonist therapy.
● With increased bleeding risk, such as but not limited to, active internal bleeding, clinically significant bleeding, bleeding at a non-compressible site, or bleeding diathesis within 30 days of randomization; platelet count less than 90,000/μL at screening; intracranial hemorrhage; major surgery, biopsy of a parenchymal organ, or serious trauma within 30 days before randomization; clinically significant gastrointestinal bleeding within 12 months before randomization; an international normalized ratio known to be higher than 1.5 at the time of screening; abciximab bolus or infusion within the preceding 8 hours, or an eptifibatide or tirofiban bolus or infusion within the past 2 hours preceding randomization; or any other condition known to increase the risk of bleeding.
● Severe concomitant condition or disease, such as cardiogenic shock at the time of randomization, ventricular arrhythmia refractory to treatment at the time of randomization, calculated creatinine clearance below 30 mL/min at screening, known significant liver disease (e.g., acute hepatitis, chronic active hepatitis, cirrhosis), or liver function test abnormalities (confirmed with repeat testing) which would require study drug discontinuation, i.e., aminoleucine transferase (ALT) more than 5 times the upper limit of the normal range (ULN) or ALT more than 3 times ULN plus total bilirubin more than 2 times ULN, prior ischemic stroke or transient ischemia attack, anemia (i.e., hemoglobin less than 10 g/ dL at screening, known clinical history of human immunodeficiency virus infection at screening, substance abuse (drug or alcohol) problem within the previous 6 months or any severe condition such as cancer that would limit life expectancy to less than 12 months.
● With an indication for long-term oral anticoagulation therapy such as atrial fibrillation, venous thromboembolism, or prior placement of a mechanical heart valve.
● With other contraindications for use of rivaroxaban and enoxaparin.
● Enrolled in another clinical study.

© 2023 Zhou S et al. *JAMA Network Open.*

eAppendix 2. Periprocedural Antithrombotic Management

All anti-thrombotic management in different clinical scenarios were used according to the guidelines for ACS managements [3-7].

(1) Patients who received thrombolytic therapy before the index event

Patients who received any thrombolytic therapy before the index event were excluded from the trial as stated in the study design.

(2) Patients with renal dysfunction

H-REPLACE trial excluded patients with severe renal dysfunction defined by creatinine clearance rate <30ml/min) and patients treated with GP IIb/IIIa inhibitors prior the enrollment. In the drug product information of enoxaparin, it is stated that: "for patients with moderate (creatinine clearance rate at 30~50 ml/min) and mild (creatinine clearance rate at 50~80 ml/min) renal dysfunction, there is no need for dose adjustment, but enoxaparin should be carefully used under intensive monitoring", so no dose adjustment was performed for patients enrolled in the enoxaparin group. For rivaroxaban, the drug product information recommends reducing the dosage for patients with creatinine clearance rate at 15-49ml/min, our trial already used the low (5 mg bid) and very low dose (2.5 mg bid) of rivaroxaban, so no dose adjustment was performed for patients enrolled to rivaroxaban groups in this trial.

(3) Pre-procedural anti-thrombotic management

All patients stopped anticoagulant drugs once (usually 12 hours before the procedure) before going to the catheter lab or surgery room.

(4) Intra-procedural anti-thrombotic management

© 2023 Zhou S et al. *JAMA Network Open.*

During PCI, intravenous enoxaparin was used for the purpose of anticoagulation, the dose was adjusted by ACT time. Other antithrombotic medications such as fondaparinux sodium and bivalirudin were not allowed to be used during the peri-PCI period.

(5) Post-procedural anti-thrombotic management

GP IIb/IIIa inhibitors were only used in patients with heavy thrombotic burden for 24 to 36 hours post PCI.

© 2023 Zhou S et al. *JAMA Network Open.*

eAppendix 3. End Point Definitions

There are two primary endpoints in this study.

1) The primary efficacy endpoint was a composite of major adverse cardiac events (MACEs), including cardiac death, myocardial infarction, re-revascularization or stroke during the 6-month follow-up period.

✧ **Cardiac death**: attribution of death to a cardiovascular etiology are acute myocardial infarction, sudden cardiac death, HF, stroke, cardiovascular procedure, cardiovascular hemorrhage, and other cardiovascular causes.

✧ **Myocardial infarction**: The categorization and definitions of the types of myocardial infarction are derived from the "Fourth Universal Definition of Myocardial Infarction", the "2014 AHA/ACC Guideline for the Management of Patients with Non-ST- Elevation Acute Coronary Syndromes", and the "2015 ACC/AHA/SCAI Guideline for the Management of ST-Elevation Myocardial Infarction".

✧ **Re-revascularization**: coronary revascularization procedures needed to treat symptoms of myocardial ischemia or based solely on coronary anatomic characteristics during the first 6 months after initial myocardial revascularization.

✧ **Stroke**: stroke is defined on the basis of the presence of acute infarction as demonstrated by imaging or based on the persistence of symptoms.

2) The primary safety endpoint was bleeding events according to the ISTH definition during the 6-month follow-up period.

Bleeding events: our prespecified analysis plan of the current trial has a schedule to report bleeding events according to the ISTH scale (major, clinically relevant non-

© 2023 Zhou S et al. *JAMA Network Open.*

major [CRNM], and minor bleeding) which is adjudicated by an independent Clinical Events Committee unaware of randomized treatment assignment. The definitions of bleeding events of ISTH scale are provided as follows in Table S1.

Cardiac-related rehospitalization: cardiovascular re-admission was defined as non-elective repeat hospitalization in all patients alive at discharge for one or more of the following: angina, MI, coronary artery bypass graft surgery (CABG), non-staged/non-index artery PCI, heart failure or stroke.

All-cause death: defined as death due to any cause.

© 2023 Zhou S et al. *JAMA Network Open.*

eAppendix 4. Standard Operation Procedure for Adjudication Process

We followed the sample pathway for the clinical events classification (CEC) process[2]. Physicians adjudicate each suspected event (also known as an event trigger) identified by either sites, programmed queries, core laboratories, or manual trigger procedures using prespecified endpoint criteria. A suspected event is allocated to two physicians acting independently. If one reviewer requests and receives additional information, this information is also distributed to the other reviewer. In a situation where the two reviewers agree in their adjudication of a suspected event, the endpoint classification is deemed complete. Otherwise, the event is usually referred to an adjudication committee. Three physicians are required for the secondary review. In this review, the decision is made by consensus.[2]

SAEs and suspected unexpected serious adverse reactions (SUSARs) should be reported only as a potential endpoint event during the adjudication process and are not infrequently obtained while reviewing a hospitalization event. These CEC-identified clinical events should be communicated to the safety surveillance/pharmacovigilance team. The process for reviewing events that are submitted by sites to the CEC Committee but then not adjudicated as an endpoint event—that is, negatively adjudicated events (NAEs)—is often not spelled out in the trial protocol, even though they may represent a source of SAEs, including SUSARs. All SAEs, including NAEs, reported by sites are to be reviewed by a safety surveillance/pharmacovigilance team, to ensure compliance with reporting requirements.

© 2023 Zhou S et al. *JAMA Network Open.*

eAppendix 5. Determination of Noninferior Margin

The noninferiority margins for primary endpoints were determined before randomization of the first patient.

To determine the non-inferiority margin of the primary efficacy endpoint, we referred to the publication of NEJM[8] (Comparison of fondaparinux and enoxaparin in acute coronary syndromes, the margin was 1.185 in this paper).

Regarding the primary safety endpoint (bleeding event), we had not found the published study with the same endpoint. We estimated the potential margin according to the “fixed margin approach” [9] recommended by FDA based on the parameter regarding major bleeding events from a meta-analysis,[10] which reported LMWH was associated with a significantly increased risk of major bleeding (OR 2.26, 95% CI 1.63–3.14); therefore, we obtained the square root of the lower bound of the 95 percent confidence interval of 1.63, that is 1.28. Moreover, a margin of (0.8, 1.25) is usually used in bioequivalence studies.[11] The non-inferiority margin of the primary safety endpoint was finally set at 1.24 in our trial with conservative clinical determination.

Both noninferior margins were determined before randomization of the first patient.

© 2023 Zhou S et al. *JAMA Network Open.*

eTable 1. Definitions of ISTH Bleeding Events

Event Classification	Definition
ISTH major bleeding	Hemoglobin drops of >2 g/dL, transfusion of >2 units packed red blood cells, symptomatic bleed in a critical area, or fatal bleeding
ISTH CRNM bleeding	Requires or prolongs hospitalization or results in laboratory testing, imaging, compression, a procedure, interruption of the study medication, or a change in concomitant therapies
ISTH Minor	Overt bleeding that does not meet criteria for CRNM or Major bleeding

© 2023 Zhou S et al. *JAMA Network Open.*

eTable 2. Baseline Characteristics (ITT Analysis)

~~a~~ Characteristic[a] ~~Patients, No. (%)~~	Patients, No. (%)		
	Enoxaparin 1 mg/kg ~~Q12H~~ (n = 682)[a]	Rivaroxaban	
		~~Rivaroxaban~~ 2.5 mg ~~twice daily~~ (n = 686)	~~Rivaroxaban~~ 5 mg ~~twice daily~~ (n = 687)
Age, mean (SD), y~~.~~	65.4 ± 8.6	66.2 ± 7.9	65.8 ± 8.1
~~Male~~Sex	~~486 (71.3)~~	~~483 (70.4)~~	~~477 (69.4)~~
Female	196 (28.7)	203 (29.6)	210 (30.6)
Male	486 (71.3)	483 (70.4)	477 (69.4)
Race and ethnicity			
Han	614 (90.0)	623 (90.8)	618 (90.0)
Tujia	43 (6.3)	390 (5.7)	41 (6.0)
Miao	22 (3.2)	21 (3.1)	26 (3.8)
other[b]	3 (0.4)	2 (0.3)	2 (0.3)
Body Mass Index, mean (SD)~~, kg/m²~~	22.85 ± 3.97	23.25 ± 4.10	22.47 ± 3.82
~~b~~ Creatinine clearance, median (IQR), mL~~l~~/min[c]	84.1 (67.4-104.8)	85.2 (68.1-106.2)	83.2 (63.1-106.4)
Smoking	162 (23.8)	160 (23.3)	158 (23.0)
Medical history			
Hypertension	434 (63.6)	426 (62.1)	419 (61.0)
Dyslipidemia	404 (59.2)	401 (58.9)	384 (55.9)
Diabetes ~~mellitus~~	231 (33.9)	216 (31.5)	231 (33.6)
Previous myocardial infarction	110 (16.1)	99 (14.4)	97 (14.1)
Previous PCI or CABG	149 (21.8)	152 (22.2)	141 (20.5)
Previous stroke	39 (5.7)	36 (5.2)	35 (5.1)
Index diagnosis			
STEMI	284 (41.6)	259 (37.8)	270 (39.3)
NSTEMI	200 (29.3)	228 (33.3)	220 (32.1)
Unstable angina	198 (29.0)	199 (29.0)	196 (28.5)
PCI or CABG for index event	485 (71.1)	490 (71.4)	482 (70.2)
PCI	476 (69.8)	484 (70.5)	474 (69.0)
CABG	9 (1.3)	6 (0.9)	8 (1.2)
Medication use			
Beta-blocker	422 (61.9)	419 (61.1)	441 (64.2)
ACE inhibitor or ARB	294 (43.1)	303 (44.1)	308 (44.9)
Statin	570 (83.6)	593 (86.4)	563 (82.0)
Calcium-channel block	147 (21.6)	127 (18.5)	161 (23.4)
Procedural aspects			

© 2023 Zhou S et al. *JAMA Network Open.*

Radial access	467 (98.1)	470 (97.2)	463 (97.7)
Clot burden (TIMI thrombus grade ≥3)	87 (18.3)	102 (21.1)	91 (19.4)
TIMI flow grade			
0	44 (9.2)	52 (10.7)	46 (9.7)
1	12 (2.5)	21 (4.3)	22 (4.6)
2	31 (6.5)	29 (6.0)	23 (4.9)
3	389 (81.7)	382 (78.9)	383 (80.8)
Duration of anticoagulation, mean (SD), (min)	52.5 ± 11.2	55.9 ± 10.8	53.4 ± 12.1
Reasons for missing opportunity of direct reperfusion			
Patient delay	173 (60.9)	158 (61.0)	180 (66.7)
Financial issues	43 (15.1)	48 (18.5)	39 (14.4)
Misdiagnosis	21 (7.4)	17 (6.5)	22 (8.1)
Others	47 (16.5)	36 (13.9)	29 (10.7)

Abbreviations: ACE, angiotensin-converting enzyme; ARB, angiotensin-receptor blocker; BMI, body mass index (calculated as weight in kilograms divided by height in meters squared); CABG, coronary artery bypass grafting; NSTEMI, non-ST -segment elevation myocardial infarction; PCI, percutaneous coronary intervention; STEMI ST-segment elevation myocardial infarction, TIMI Thrombolysis in Myocardial Infarction.

[a]There were no significant differences among the three groups.

[b]other ethnic minority (e.g., Hui, Mongol)

[c]Creatinine clearance was calculated with the Cockcroft–Gault equation.

[a] Variables expressed as mean ± (SD), median (25th percentile–75th percentile), or percentage. There were no significant differences among the three groups. ACE denotes angiotensin-converting enzyme, ARB angiotensin-receptor blocker, CABG coronary artery bypass grafting, NSTEMI, non-ST-segment elevation myocardial infarction, PCI percutaneous coronary intervention, Q12H every 12 hours, STEMI, ST-segment elevation myocardial infarction, TIMI Thrombolysis in Myocardial Infarction.

[b] Creatinine clearance was calculated with the Cockcroft–Gault equation.

© 2023 Zhou S et al. *JAMA Network Open*.

eTable 3. Primary and Secondary End Points (ITT Analysis)

**End points**	Enoxaparin 1 mg/kg Q12H	Rivaroxaban 2.5 mg twice daily	Rivaroxaban 5 mg twice daily		Rivaroxaban 2.5 mg vs. Enoxaparin			Rivaroxaban 5 mg vs. Enoxaparin	
	n=682	n=686	n=687		Hazard Ratio (95% CI)	*P* value*		Hazard Ratio (95% CI)	*P* value*
**Safety**									
Primary safety endpoint —any bleeding	46 (6.74)	32 (4.66)	36 (5.24)		0.68 (0.43-1.07)	0.008[a]		0.76 (0.49-1.18)	0.02[b]
ISTH major bleeding	8 (1.17)	5 (0.73)	5 (0.73)		0.62 (0.20-1.89)			0.62 (0.20-1.89)	
ISTH CRNM bleeding	9 (1.32)	8 (1.17)	8 (1.16)		0.88 (0.34-2.29)			0.88 (0.34-2.28)	
ISTH minor bleeding	29 (4.25)	19 (2.77)	23 (3.35)		0.64 (0.36-1.14)			0.78 (0.45-1.35)	
**Efficacy**									
Primary efficacy endpoint— Composite endpoint of cardiac death, myocardial infarction, re-revascularization or stroke	23 (3.37)	16 (2.33)	14 (2.04)		0.68 (0.36-1.29)	0.05[d]		0.60 (0.31-1.16)	0.02[c]
Cardiac death	3 (0.44)	2 (0.29)	2 (0.29)		0.66 (0.11-3.98)			0.67 (0.11-3.97)	
Myocardial infarction	9 (1.32)	6 (0.87)	5 (0.73)		0.66 (0.23-1.85)			0.55 (0.18-1.64)	
Re-revascularization	3 (0.44)	2 (0.29)	2 (0.29)		0.66 (0.11-3.96)			0.66 (0.11-3.95)	
Stroke									

© 2023 Zhou S et al. *JAMA Network Open.*

Any	8 (1.17)	6 (0.87)	5 (0.73)		0.74 (0.26-2.14)			0.62 (0.20-1.89)	
Ischemic	4 (0.59)	2 (0.29)	3 (0.44)		0.50 (0.09-2.70)			0.74 (0.17-3.32)	
Secondary efficacy endpoint									
All-cause death	5 (0.73)	3 (0.44)	4 (0.58)		0.60 (0.14-2.49)			0.79 (0.21-2.95)	
Cardiac-related rehospitalization	56 (8.21)	49 (7.14)	51 (7.42)		0.85 (0.58-1.25)			0.89 (0.61-1.30)	

* Primary endpoints were tested sequentially (from [a] to [d]). 1-sided P value was statistically significant if $P<0.025$ for each step. Data for the endpoints correspond to the intention-to-treat (ITT) analysis. The hazard ratios and 95% CIs were calculated by Cox proportional hazards analysis, and the P values for noninferiority were calculated by PROBNORM[(estimate-LN(L))/SE)], where PROBNORM is standard normal distribution function, estimate = Parameter estimate, L= Margin; SE = Standard Error. For secondary endpoints, the common two-sided tests with $\alpha = 0.05$ were used. CRNM, clinically relevant non-major; Q12H, every 12 hours.

© 2023 Zhou S et al. *JAMA Network Open.*

eFigure 1. Risk of Primary Safety End Point Based on Major Subgroup

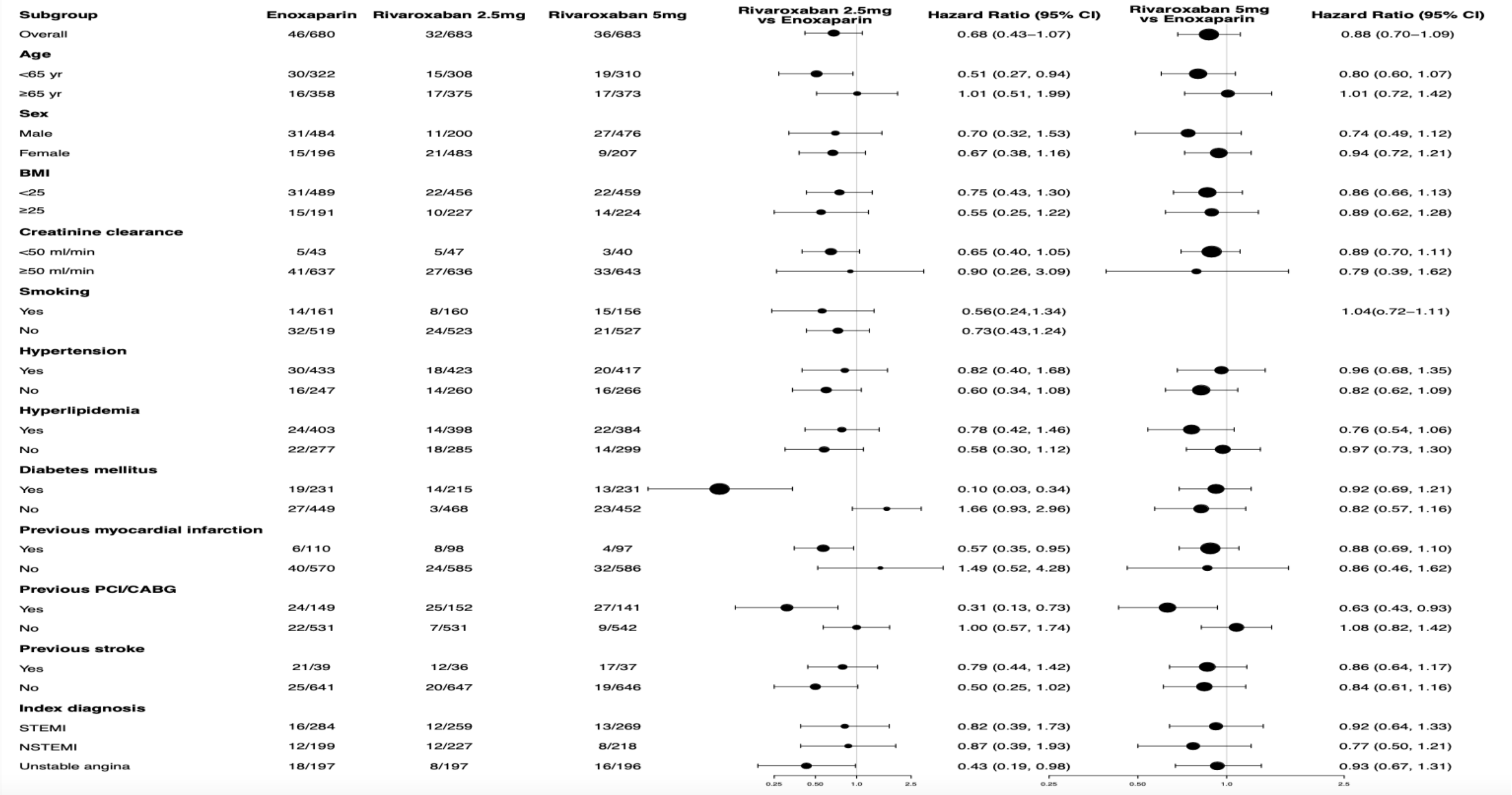

Subgroup	Enoxaparin	Rivaroxaban 2.5mg	Rivaroxaban 5mg	Rivaroxaban 2.5mg vs Enoxaparin	Hazard Ratio (95% CI)	Rivaroxaban 5mg vs Enoxaparin	Hazard Ratio (95% CI)
Overall	46/680	32/683	36/683		0.68 (0.43–1.07)		0.88 (0.70–1.09)
**Age**							
<65 yr	30/322	15/308	19/310		0.51 (0.27, 0.94)		0.80 (0.60, 1.07)
≥65 yr	16/358	17/375	17/373		1.01 (0.51, 1.99)		1.01 (0.72, 1.42)
**Sex**							
Male	31/484	11/200	27/476		0.70 (0.32, 1.53)		0.74 (0.49, 1.12)
Female	15/196	21/483	9/207		0.67 (0.38, 1.16)		0.94 (0.72, 1.21)
**BMI**							
<25	31/489	22/456	22/459		0.75 (0.43, 1.30)		0.86 (0.66, 1.13)
≥25	15/191	10/227	14/224		0.55 (0.25, 1.22)		0.89 (0.62, 1.28)
**Creatinine clearance**							
<50 ml/min	5/43	5/47	3/40		0.65 (0.40, 1.05)		0.89 (0.70, 1.11)
≥50 ml/min	41/637	27/636	33/643		0.90 (0.26, 3.09)		0.79 (0.39, 1.62)
**Smoking**							
Yes	14/161	8/160	15/156		0.56(0.24,1.34)		1.04(o.72–1.11)
No	32/519	24/523	21/527		0.73(0.43,1.24)		
**Hypertension**							
Yes	30/433	18/423	20/417		0.82 (0.40, 1.68)		0.96 (0.68, 1.35)
No	16/247	14/260	16/266		0.60 (0.34, 1.08)		0.82 (0.62, 1.09)
**Hyperlipidemia**							
Yes	24/403	14/398	22/384		0.78 (0.42, 1.46)		0.76 (0.54, 1.06)
No	22/277	18/285	14/299		0.58 (0.30, 1.12)		0.97 (0.73, 1.30)
**Diabetes mellitus**							
Yes	19/231	14/215	13/231		0.10 (0.03, 0.34)		0.92 (0.69, 1.21)
No	27/449	3/468	23/452		1.66 (0.93, 2.96)		0.82 (0.57, 1.16)
**Previous myocardial infarction**							
Yes	6/110	8/98	4/97		0.57 (0.35, 0.95)		0.88 (0.69, 1.10)
No	40/570	24/585	32/586		1.49 (0.52, 4.28)		0.86 (0.46, 1.62)
**Previous PCI/CABG**							
Yes	24/149	25/152	27/141		0.31 (0.13, 0.73)		0.63 (0.43, 0.93)
No	22/531	7/531	9/542		1.00 (0.57, 1.74)		1.08 (0.82, 1.42)
**Previous stroke**							
Yes	21/39	12/36	17/37		0.79 (0.44, 1.42)		0.86 (0.64, 1.17)
No	25/641	20/647	19/646		0.50 (0.25, 1.02)		0.84 (0.61, 1.16)
**Index diagnosis**							
STEMI	16/284	12/259	13/269		0.82 (0.39, 1.73)		0.92 (0.64, 1.33)
NSTEMI	12/199	12/227	8/218		0.87 (0.39, 1.93)		0.77 (0.50, 1.21)
Unstable angina	18/197	8/197	16/196		0.43 (0.19, 0.98)		0.93 (0.67, 1.31)

The black boxes represent the relative risk with 95% CIs (horizontal lines). HRs and 95% CIs were from Cox proportional hazard models with

© 2023 Zhou S et al. *JAMA Network Open.*

subgroups. BMI, body mass index; STEMI, ST segment elevation myocardial infarction; NSTEMI, non-ST segment elevation myocardial infarction; PCI, percutaneous coronary intervention; and CABG, coronary artery bypass graft.

© 2023 Zhou S et al. *JAMA Network Open.*

eFigure 2. Risk of Primary Efficacy End Point Based on Major Subgroup

Subgroup	Enoxaparin	Rivaroxaban 2.5mg	Rivaroxaban 5mg	Rivaroxaban 2.5mg vs Enoxaparin Hazard Ratio (95% CI)	Rivaroxaban 5mg vs Enoxaparin Hazard Ratio (95% CI)
Overall	23/680	16/683	14/683	0.68 (0.36–1.30)	0.60 (0.31–1.16)
**Age**					
<65 yr	15/322	4/308	8/310	0.27 (0.09, 0.82)	0.74 (0.48, 1.13)
≥65 yr	8/358	12/375	6/373	1.43 (0.58, 3.50)	0.85 (0.50, 1.44)
**Sex**					
Male	11/484	8/483	9/476	0.64 (0.26, 1.56)	0.62 (0.37, 1.05)
Female	12/196	8/200	5/207	0.72 (0.29, 1.80)	0.91 (0.58, 1.41)
**BMI**					
<25	15/489	6/227	10/459	0.71 (0.32, 1.58)	0.84 (0.56, 1.25)
≥25	8/191	10/456	4/224	0.62 (0.21, 1.78)	0.65 (0.35, 1.18)
**Creatinine clearance**					
<50 ml/min	22/637	3/47	3/40	0.58 (0.29, 1.16)	0.70 (0.49, 1.00)
≥50 ml/min	1/43	13/636	11/643	2.80 (0.29, 26.96)	1.82 (0.59, 5.64)
**Smoking**					
Yes	4/161	2/160	4/157	0.72(0.36, 1.21)	1.01(0.50–2.02)
No	19/519	14/523	10/526	0.50(0.09,2.74)	0.71(0.49,1.05)
**Hypertension**					
Yes	14/433	10/423	8/417	0.62 (0.22, 1.75)	0.78 (0.47, 1.31)
No	9/247	6/260	6/266	0.72 (0.32, 1.62)	0.77 (0.50, 1.18)
**Hyperlipidemia**					
Yes	8/403	5/398	7/384	0.70 (0.32, 1.52)	0.65 (0.42, 1.02)
No	15/277	11/285	7/299	0.63 (0.21, 1.92)	0.95 (0.57, 1.59)
**Diabetes mellitus**					
Yes	10/231	12/215	6/231	0.29 (0.10, 0.89)	0.78 (0.50, 1.21)
No	13/449	4/468	8/452	1.28 (0.55, 2.96)	0.77 (0.46, 1.28)
**Previous myocardial infarction**					
Yes	2/110	4/98	2/97	0.55 (0.27, 1.11)	0.74 (0.52, 1.06)
No	21/570	12/585	12/586	2.26 (0.41, 12.35)	1.06 (0.40, 2.83)
**Previous PCI/CABG**					
Yes	12/149	12/152	10/141	0.36 (0.11, 1.13)	0.59 (0.34, 1.05)
No	11/531	4/531	4/542	0.95 (0.43, 2.12)	0.93 (0.61, 1.41)
**Previous stroke**					
Yes	14/39	8/36	7/37	0.88 (0.34, 2.28)	0.88 (0.54, 1.44)
No	9/641	8/647	7/646	0.50 (0.21, 1.19)	0.66 (0.42, 1.04)
**Index diagnosis**					
STEMI	5/284	4/259	5/269	0.87 (0.23, 3.24)	1.03 (0.55, 1.91)
NSTEMI	6/199	10/227	3/218	1.47 (0.53, 4.03)	0.67 (0.34, 1.35)
Unstable angina	12/197	2/197	8/487	0.16 (0.04, 0.72)	0.70 (0.43, 1.14)

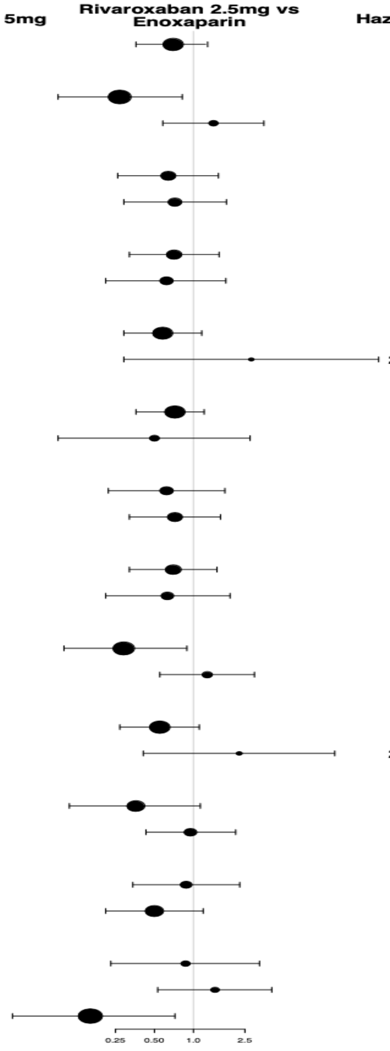

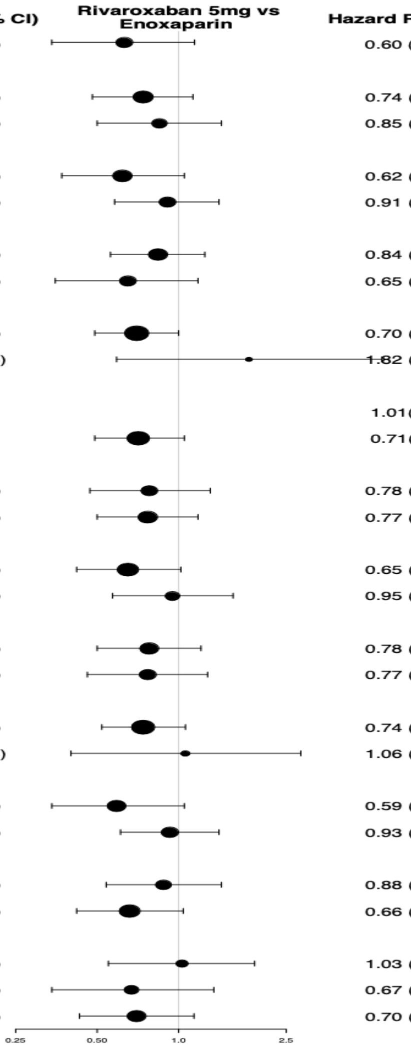

© 2023 Zhou S et al. *JAMA Network Open.*

The black boxes represent the relative risk and 95% CIs (horizontal lines). HRs and 95% CIs were calculated from Cox proportional hazard models within the subgroups. BMI, body mass index; STEMI, ST segment elevation myocardial infarction; NSTEMI, non-ST segment elevation myocardial infarction; PCI, percutaneous coronary intervention; and CABG, coronary artery bypass graft.

© 2023 Zhou S et al. *JAMA Network Open.*

eReferences.

1. Zhou S. Safety and Efficacy of LMWH Versus Rivaroxaban in Chinese Patients Hospitalized With Acute Coronary Syndrome. ClinicalTrials.gov. December 5, 2017. Accessed April 8, 2022. https://clinicaltrials.gov/ct2/show/NCT03363035.
2. Lopes RD, Dickerson S, Hafley G, et al. Methodology of a reevaluation of cardiovascular outcomes in the RECORD trial: study design and conduct. Am Heart J. 2013;166(2):208-216.e28.
3. Amsterdam EA, Wenger NK, Brindis RG, et al. 2014 AHA/ACC guideline for the management of patients with non-ST-elevation acute coronary syndromes: executive summary: a report of the American College of Cardiology/American Heart Association Task Force on Practice Guidelines. Circulation. 2014;130(25):2354-2394.
4. Levine GN, Bates ER, Blankenship JC, et al. 2015 ACC/AHA/SCAI Focused Update on Primary Percutaneous Coronary Intervention for Patients With ST-Elevation Myocardial Infarction: An Update of the 2011 ACCF/AHA/SCAI Guideline for Percutaneous Coronary Intervention and the 2013 ACCF/AHA Guideline for the Management of ST-Elevation Myocardial Infarction. J Am Coll Cardiol. 2016;67(10):1235-1250.
5. Roffi M, Patrono C, Collet JP, et al. 2015 ESC Guidelines for the management of acute coronary syndromes in patients presenting without persistent ST-segment elevation: Task Force for the Management of Acute Coronary Syndromes in Patients Presenting without Persistent ST-Segment Elevation of the European Society of Cardiology (ESC). Eur Heart J. 2016;37(3):267-315.

© 2023 Zhou S et al. *JAMA Network Open.*

6. Ibanez B, James S, Agewall S, et al. 2017 ESC Guidelines for the management of acute myocardial infarction in patients presenting with ST-segment elevation: The Task Force for the management of acute myocardial infarction in patients presenting with ST-segment elevation of the European Society of Cardiology (ESC). Eur Heart J. 2018;39(2):119-177.
7. Section of Interventional Cardiology of Chinese Society of Cardiology of Chinese Medical Association, Specialty Committee on Prevention and Treatment of Thrombosis of Chinese College of Cardiovascular Physicians, Editorial Board of Chinese Journal of Cardiology. Chinese guideline for percutaneous coronary intervention (2016). Zhonghua Xin Xue Guan Bing Za Zhi. 2016;44(5):382-400. doi: 10.3760/cma.j.issn.0253-3758.2016.05.006.
8. Fifth Organization to Assess Strategies in Acute Ischemic Syndromes Investigators, Yusuf Salim, Mehta Shamir R, et al. Comparison of fondaparinux and enoxaparin in acute coronary syndromes. N Engl J Med. 2006;354(14): 1464-1476.
9. US Department of Health and Human Services, US Food and Drug Administration, Center for Biologics Evaluation and Research (CBER). Guidance for industry non-inferiority clinical trials. Published March 2010. Accessed April 8,2022.www.fda.gov/downloads/drugs/guidancecomplianceregulatoryinformation/guidances/ucm202140.pdf.
10. Eikelboom JW, Anand SS, Malmberg K, Weitz JI, Ginsberg JS, Yusuf S. Unfractionated heparin and low-molecular-weight heparin in acute coronary syndrome without ST elevation: a meta-analysis. Lancet.

2000;355(9219):1936-1942.

11. U.S. Department of Health and Human Services, Food and Drug Administration, Center for Drug Evaluation and Research (CDER). Guidance for industry: statistical Approaches to Establishing Bioequivalence. Published April 29, 2020. Accessed April 8, 2022. http://www.fda.gov/downloads/drugs/guidancecomplianceregulatoryinformation/guidances/ucm070244.pdf.

© 2023 Zhou S et al. *JAMA Network Open.*
