# Supplementary material for: Effect of Rivaroxaban vs Enoxaparin on Major Cardiac Adverse Events and Bleeding Risk in the Acute Phase of Acute Coronary Syndrome: The H-REPLACE Randomized Equivalence and Noninferiority Trial
Source: JAMA Netw Open. 2023 Feb 10;6(2):e2255709. doi: 10.1001/jamanetworkopen.2022.55709 (PMC9918885; doi:10.1001/jamanetworkopen.2022.55709)
Supplement: Supplement 3. — The H-REPLACE Investigators [file jamanetwopen-e2255709-s003.pdf]

Supplemental Online Content: Nonauthor Collaborators
*First name, last name, and suffix (if applicable) are required and will appear in PubMed.

***Group Name(s): the H-REPLACE Investigators**

***First Name and Middle Initial(s)**	***Last Name**	***Suffix (eg, Jr, III)**	Academic Degrees	Institution	Location (city, state/province, country)	Role or Contribution, eg, chair, principal investigator	Group (if more than 1 Group listed in the byline) and/or Subgroup (eg, Steering Committee)
Xinqun	Hu		MD	The Second Xiangya Hospital of Central South University	Changsha, Hunan, China	Screening and enrollment	the H-REPLACE Investigators
Zhenfei	Fang		MD	The Second Xiangya Hospital of Central South University	Changsha, Hunan, China	Screening and enrollment	the H-REPLACE Investigators
Liang	Tang		MD	The Second Xiangya Hospital of Central South University	Changsha, Hunan, China	Screening and enrollment	the H-REPLACE Investigators
Xuping	Li		MD	The Second Xiangya Hospital of Central South University	Changsha, Hunan, China	Screening and enrollment	the H-REPLACE Investigators
Zhaowei	Zhu		MD	The Second Xiangya Hospital of Central South University	Changsha, Hunan, China	Screening and enrollment	the H-REPLACE Investigators
Shi	Tai		MD	The Second Xiangya Hospital of Central South University	Changsha, Hunan, China	Screening and enrollment	the H-REPLACE Investigators
Tao	Tu		MD	The Second Xiangya Hospital of Central South University	Changsha, Hunan, China	Screening and enrollment	the H-REPLACE Investigators
Hui	Yang		MD	The Second Xiangya Hospital of Central South University	Changsha, Hunan, China	Screening and enrollment	the H-REPLACE Investigators
Yuhu	He		MD	The Second Xiangya Hospital of Central South University	Changsha, Hunan, China	Screening and enrollment	the H-REPLACE Investigators
Mingxian	Chen		MD	The Second Xiangya Hospital of Central South University	Changsha, Hunan, China	Screening and enrollment	the H-REPLACE Investigators
Daoquan	Peng		MD	The Second Xiangya Hospital of Central South University	Changsha, Hunan, China	Screening and enrollment	the H-REPLACE Investigators
Yanshu	Zhao		MD	The Second Xiangya Hospital of Central South University	Changsha, Hunan, China	Screening and enrollment	the H-REPLACE Investigators
Danyan	Xu		MD	The Second Xiangya Hospital of Central South University	Changsha, Hunan, China	Screening and enrollment	the H-REPLACE Investigators
Jianjun	Tang		MD	The Second Xiangya Hospital of Central South University	Changsha, Hunan, China	Screening and enrollment	the H-REPLACE Investigators
Jiang	Li		MD	The Second Xiangya Hospital of Central South University	Changsha, Hunan, China	Screening and enrollment	the H-REPLACE Investigators

Supplemental Online Content: Nonauthor Collaborators
*First name, last name, and suffix (if applicable) are required and will appear in PubMed.

***First Name and Middle Initial(s)**	***Last Name**	***Suffix (eg, Jr, III)**	Academic Degrees	Institution	Location (city, state/province, country)	Role or Contribution, eg, chair, principal investigator	Group (if more than 1 Group listed in the byline) and/or Subgroup (eg, Steering Committee)
Yaqin	Chen		MD	The Second Xiangya Hospital of Central South University	Changsha, Hunan, China	Screening and enrollment	the H-REPLACE Investigators
Yi	Li		MM	The Second Xiangya Hospital of Central South University	Changsha, Hunan, China	data collection	the H-REPLACE Investigators
Xiaolan	Luo		MD	The Second Xiangya Hospital of Central South University	Changsha, Hunan, China	Screening and enrollment	the H-REPLACE Investigators
Yong	Huang		MM	Xiangxiang People's Hospital	Xiangtan, Hunan, China	data collection	the H-REPLACE Investigators
Jianqiang	Xiang		MM	Xiangxiang People's Hospital	Xiangtan, Hunan, China	Screening and enrollment	the H-REPLACE Investigators
Qinglin	Guan		MM	Xiangxiang People's Hospital	Xiangtan, Hunan, China	Screening and enrollment	the H-REPLACE Investigators
Xinyang	Li		MM	Xiangxiang People's Hospital	Xiangtan, Hunan, China	Screening and enrollment	the H-REPLACE Investigators
Xiaojun	Yi		MM	Xiangxiang People's Hospital	Xiangtan, Hunan, China	Screening and enrollment	the H-REPLACE Investigators
Fang	Tan		MM	Xiangxiang People's Hospital	Xiangtan, Hunan, China	Screening and enrollment	the H-REPLACE Investigators
Yan	Liu		MM	Xiangxiang People's Hospital	Xiangtan, Hunan, China	Screening and enrollment	the H-REPLACE Investigators
Jiang	Pan		MM	Xiangxiang People's Hospital	Xiangtan, Hunan, China	Screening and enrollment	the H-REPLACE Investigators
Jianqiang	Peng		MD	Hunan Provincial People's Hospital, The First Affiliated Hospital of Hunan Normal University	Changsha, Hunan, China	Screening and enrollment	the H-REPLACE Investigators
Sulan	Yan		MM	Hunan Provincial People's Hospital, The First Affiliated Hospital of Hunan Normal University	Changsha, Hunan, China	Screening and enrollment	the H-REPLACE Investigators
Zhengyu	Liu		MM	Hunan Provincial People's Hospital, The First Affiliated Hospital of Hunan Normal University	Changsha, Hunan, China	Screening and enrollment	the H-REPLACE Investigators

Supplemental Online Content: Nonauthor Collaborators
*First name, last name, and suffix (if applicable) are required and will appear in PubMed.

*First Name and Middle Initial(s)	*Last Name	*Suffix (eg, Jr, III)	Academic Degrees	Institution	Location (city, state/province, country)	Role or Contribution, eg, chair, principal investigator	Group (if more than 1 Group listed in the byline) and/or Subgroup (eg, Steering Committee)
Bo	Cui		MM	Hunan Provincial People's Hospital, The First Affiliated Hospital of Hunan Normal University	Changsha, Hunan, China	Screening and enrollment	the H-REPLACE Investigators
Peng	Wang		MM	Hunan Provincial People's Hospital, The First Affiliated Hospital of Hunan Normal University	Changsha, Hunan, China	Screening and enrollment	the H-REPLACE Investigators
Yi	Tang		MD	Hunan Provincial People's Hospital, The First Affiliated Hospital of Hunan Normal University	Changsha, Hunan, China	Screening and enrollment	the H-REPLACE Investigators
Juan	Wen		MM	The Third Xiangya Hospital of Central South University Changsha	Changsha, Hunan, China	data collection	the H-REPLACE Investigators
Hong	Liu		MM	The Third Xiangya Hospital of Central South University Changsha	Changsha, Hunan, China	Screening and enrollment	the H-REPLACE Investigators
Chunyan	Weng		MM	The Third Xiangya Hospital of Central South University Changsha	Changsha, Hunan, China	Screening and enrollment	the H-REPLACE Investigators
Xi	Wu		MM	The Third Xiangya Hospital of Central South University Changsha	Changsha, Hunan, China	Screening and enrollment	the H-REPLACE Investigators
Jingjia	Yu		MM	The Third Xiangya Hospital of Central South University Changsha	Changsha, Hunan, China	Screening and enrollment	the H-REPLACE Investigators
Qiong	Yang		MM	The Third Xiangya Hospital of Central South University Changsha	Changsha, Hunan, China	Screening and enrollment	the H-REPLACE Investigators
Liping	Peng		MM	The Third Xiangya Hospital of Central South University Changsha	Changsha, Hunan, China	Screening and enrollment	the H-REPLACE Investigators

Supplemental Online Content: Nonauthor Collaborators
*First name, last name, and suffix (if applicable) are required and will appear in PubMed.

***First Name and Middle Initial(s)**	***Last Name**	***Suffix (eg, Jr, III)**	Academic Degrees	Institution	Location (city, state/province, country)	Role or Contribution, eg, chair, principal investigator	Group (if more than 1 Group listed in the byline) and/or Subgroup (eg, Steering Committee)
Anying	Li		MM	The Third Xiangya Hospital of Central South University Changsha	Changsha, Hunan, China	Screening and enrollment	the H-REPLACE Investigators
Zhihui	Zhang			The Third Xiangya Hospital of Central South University Changsha	Changsha, Hunan, China	Screening and enrollment	the H-REPLACE Investigators
Xiaowei	Zhou		MM	The Third Xiangya Hospital of Central South University Changsha	Changsha, Hunan, China	Screening and enrollment	the H-REPLACE Investigators
Huaqing	Tan		MD	The First People's Hospital of Loudi	Loudi, Hunan, China	Sub-investigator	the H-REPLACE Investigators
Jie	Chen		MM	The First People's Hospital of Loudi	Loudi, Hunan, China	data collection	the H-REPLACE Investigators
Zhiyuan	Yang		MM	The First People's Hospital of Loudi	Loudi, Hunan, China	Screening and enrollment	the H-REPLACE Investigators
Zhizhong	Liang		MM	The First People's Hospital of Loudi	Loudi, Hunan, China	Screening and enrollment	the H-REPLACE Investigators
Xiangyu	Tang		MM	The First People's Hospital of Loudi	Loudi, Hunan, China	Screening and enrollment	the H-REPLACE Investigators
Sha	Chen		MM	The First People's Hospital of Loudi	Loudi, Hunan, China	Screening and enrollment	the H-REPLACE Investigators
Lan	Zeng		MM	The First People's Hospital of Loudi	Loudi, Hunan, China	Screening and enrollment	the H-REPLACE Investigators
Yingtao	Wu		MM	The First People's Hospital of Loudi	Loudi, Hunan, China	Screening and enrollment	the H-REPLACE Investigators
Guangyao	Chen		MM	The First People's Hospital of Loudi	Loudi, Hunan, China	Screening and enrollment	the H-REPLACE Investigators
Dihua	Yan		MM	The First People's Hospital of Loudi	Loudi, Hunan, China	Screening and enrollment	the H-REPLACE Investigators
Lianghui	Zhou		MM	The First People's Hospital of Loudi	Loudi, Hunan, China	Screening and enrollment	the H-REPLACE Investigators
Siqin	Yuan		MM	The First People's Hospital of Loudi	Loudi, Hunan, China	Screening and enrollment	the H-REPLACE Investigators

Supplemental Online Content: Nonauthor Collaborators
*First name, last name, and suffix (if applicable) are required and will appear in PubMed.

***First Name and Middle Initial(s)**	***Last Name**	***Suffix (eg, Jr, III)**	Academic Degrees	Institution	Location (city, state/province, country)	Role or Contribution, eg, chair, principal investigator	Group (if more than 1 Group listed in the byline) and/or Subgroup (eg, Steering Committee)
Rong	Wu		MM	The First People's Hospital of Loudi	Loudi, Hunan, China	Screening and enrollment	the H-REPLACE Investigators
Pei	Zhou		MM	The First People's Hospital of Loudi	Loudi, Hunan, China	Screening and enrollment	the H-REPLACE Investigators
Yuzhen	Fu		MM	The First People's Hospital of Loudi	Loudi, Hunan, China	Screening and enrollment	the H-REPLACE Investigators
Youliang	Huang		MD	The First Affiliated Hospital of Hunan University of Medicine	Huaihua, Hunan,China	Sub-investigator	the H-REPLACE Investigators
Wei	Tang		MD	The First Affiliated Hospital of Hunan University of Medicine	Huaihua, Hunan,China	data collection	the H-REPLACE Investigators
Yang	Nie		MD	The First Affiliated Hospital of Hunan University of Medicine	Huaihua, Hunan,China	Screening and enrollment	the H-REPLACE Investigators
Hongju	Xiang		MM	The First Affiliated Hospital of Jishou University	Jishou, Hunan, China	Screening and enrollment	the H-REPLACE Investigators
Wei	Chen		MM	The First Affiliated Hospital of Jishou University	Jishou, Hunan, China	Screening and enrollment	the H-REPLACE Investigators
Zhiyong	Yang		MM	The First Affiliated Hospital of Jishou University	Jishou, Hunan, China	Screening and enrollment	the H-REPLACE Investigators
Yue	Wu		MM	The First Affiliated Hospital of Jishou University	Jishou, Hunan, China	Screening and enrollment	the H-REPLACE Investigators
Haishui	Deng		MM	The First Affiliated Hospital of Jishou University	Jishou, Hunan, China	Screening and enrollment	the H-REPLACE Investigators
Ming	Lei		MM	The First Affiliated Hospital of Jishou University	Jishou, Hunan, China	Screening and enrollment	the H-REPLACE Investigators
Bailing	Zhang		MM	The First Affiliated Hospital of Jishou University	Jishou, Hunan, China	Screening and enrollment	the H-REPLACE Investigators
Xing	Xiang		MM	The First Affiliated Hospital of Jishou University	Jishou, Hunan, China	Screening and enrollment	the H-REPLACE Investigators
Bingqing	Xue		MM	The First Affiliated Hospital of Jishou University	Jishou, Hunan, China	Screening and enrollment	the H-REPLACE Investigators
Liping	Xiong		MM	The First Affiliated Hospital of Jishou University	Jishou, Hunan, China	Screening and enrollment	the H-REPLACE Investigators

Supplemental Online Content: Nonauthor Collaborators
*First name, last name, and suffix (if applicable) are required and will appear in PubMed.

*First Name and Middle Initial(s)	*Last Name	*Suffix (eg, Jr, III)	Academic Degrees	Institution	Location (city, state/province, country)	Role or Contribution, eg, chair, principal investigator	Group (if more than 1 Group listed in the byline) and/or Subgroup (eg, Steering Committee)
Xiping	Xu		MD	The First People's Hospital of Yueyang	Yueyang, Hunan, China	Sub-investigator	the H-REPLACE Investigators
Xiaojian	Feng		MM	The First People's Hospital of Yueyang	Yueyang, Hunan, China	data collection	the H-REPLACE Investigators
Jianhua	Zhao		MM	The First People's Hospital of Yueyang	Yueyang, Hunan, China	Screening and enrollment	the H-REPLACE Investigators
Yonghua	Lu		MM	The First People's Hospital of Yueyang	Yueyang, Hunan, China	Screening and enrollment	the H-REPLACE Investigators
Zhengzai	Li		MM	The First People's Hospital of Yueyang	Yueyang, Hunan, China	Screening and enrollment	the H-REPLACE Investigators
Bo	Zhou		MM	The First People's Hospital of Yueyang	Yueyang, Hunan, China	Screening and enrollment	the H-REPLACE Investigators
Cheng	Li		MM	The First People's Hospital of Yueyang	Yueyang, Hunan, China	Screening and enrollment	the H-REPLACE Investigators
Zibin	Deng		MM	The First People's Hospital of Yueyang	Yueyang, Hunan, China	Screening and enrollment	the H-REPLACE Investigators
Hongda	Feng		MM	The First People's Hospital of Yueyang	Yueyang, Hunan, China	Screening and enrollment	the H-REPLACE Investigators
Xibin	Zhang		MM	The First People's Hospital of Yueyang	Yueyang, Hunan, China	Screening and enrollment	the H-REPLACE Investigators
Guoli	Wang		MM	The First People's Hospital of Yueyang	Yueyang, Hunan, China	Screening and enrollment	the H-REPLACE Investigators
Zhi	Chen		MM	The First People's Hospital of Yueyang	Yueyang, Hunan, China	Screening and enrollment	the H-REPLACE Investigators
Wenxuan	Zeng		MM	The First People's Hospital of Yueyang	Yueyang, Hunan, China	Screening and enrollment	the H-REPLACE Investigators
Di	Shen		MM	The First People's Hospital of Yueyang	Yueyang, Hunan, China	Screening and enrollment	the H-REPLACE Investigators
Xiangyong	Hu		MM	The First Affiliated Hospital of Jishou University	Jishou, Hunan, China	Screening and enrollment	the H-REPLACE Investigators
Xuemei	Chen		MM	The First People's Hospital of Changde City	Changde, Hunan, China	data collection	the H-REPLACE Investigators

Supplemental Online Content: Nonauthor Collaborators
*First name, last name, and suffix (if applicable) are required and will appear in PubMed.

***First Name and Middle Initial(s)**	***Last Name**	***Suffix (eg, Jr, III)**	Academic Degrees	Institution	Location (city, state/province, country)	Role or Contribution, eg, chair, principal investigator	Group (if more than 1 Group listed in the byline) and/or Subgroup (eg, Steering Committee)
Zuoan	Qin		MM	The First People's Hospital of Changde City	Changde, Hunan, China	Screening and enrollment	the H-REPLACE Investigators
Sulan	Huang		MM	The First People's Hospital of Changde City	Changde, Hunan, China	Screening and enrollment	the H-REPLACE Investigators
Ning	Guo		MM	The First People's Hospital of Changde City	Changde, Hunan, China	Screening and enrollment	the H-REPLACE Investigators
Zhixiang	Zhang		MM	The First People's Hospital of Changde City	Changde, Hunan, China	Screening and enrollment	the H-REPLACE Investigators
Kun	Chen		MM	The First People's Hospital of Changde City	Changde, Hunan, China	Screening and enrollment	the H-REPLACE Investigators
Bingxing	Gong		MM	The First People's Hospital of Changde City	Changde, Hunan, China	Screening and enrollment	the H-REPLACE Investigators
Min	Yin		MM	The First People's Hospital of Changde City	Changde, Hunan, China	Screening and enrollment	the H-REPLACE Investigators
Tao	Qin		MM	The First People's Hospital of Changde City	Changde, Hunan, China	Screening and enrollment	the H-REPLACE Investigators
Tao	Hu		MM	The First People's Hospital of Changde City	Changde, Hunan, China	Screening and enrollment	the H-REPLACE Investigators
Jiangbiao	Yu		MM	The First People's Hospital of Changde City	Changde, Hunan, China	Screening and enrollment	the H-REPLACE Investigators
Ting	Zhang		MM	The First People's Hospital of Changde City	Changde, Hunan, China	Screening and enrollment	the H-REPLACE Investigators
Xingcheng	Guo		MM	The First People's Hospital of Changde City	Changde, Hunan, China	Screening and enrollment	the H-REPLACE Investigators
Hui	Peng		MM	The First People's Hospital of Changde City	Changde, Hunan, China	Screening and enrollment	the H-REPLACE Investigators
Minming	Yin		MM	The First People's Hospital of Changde City	Changde, Hunan, China	Screening and enrollment	the H-REPLACE Investigators
Huaineng	Zhou		MM	The First People's Hospital of Changde City	Changde, Hunan, China	Screening and enrollment	the H-REPLACE Investigators
Li	Luo		MM	The First People's Hospital of Changde City	Changde, Hunan, China	Screening and enrollment	the H-REPLACE Investigators
Xiaoxia	Xiao		MM	The Third Hospital of Changsha	Changsha, Hunan, China	data collection	the H-REPLACE Investigators

Supplemental Online Content: Nonauthor Collaborators
*First name, last name, and suffix (if applicable) are required and will appear in PubMed.

***First Name and Middle Initial(s)**	***Last Name**	***Suffix (eg, Jr, III)**	Academic Degrees	Institution	Location (city, state/province, country)	Role or Contribution, eg, chair, principal investigator	Group (if more than 1 Group listed in the byline) and/or Subgroup (eg, Steering Committee)
Yong	Quan		MM	The Third Hospital of Changsha	Changsha, Hunan, China	Screening and enrollment	the H-REPLACE Investigators
Weifen	Liang		MM	The Third Hospital of Changsha	Changsha, Hunan, China	Screening and enrollment	the H-REPLACE Investigators
Yang	Wu		MM	The Third Hospital of Changsha	Changsha, Hunan, China	Screening and enrollment	the H-REPLACE Investigators
Hang	Chen		MM	The Third Hospital of Changsha	Changsha, Hunan, China	Screening and enrollment	the H-REPLACE Investigators
Wei	Shi		MM	The Third Hospital of Changsha	Changsha, Hunan, China	Screening and enrollment	the H-REPLACE Investigators
Aiying	Liu		MM	The Third Hospital of Changsha	Changsha, Hunan, China	Screening and enrollment	the H-REPLACE Investigators
Yijian	Liu		MM	The Third Hospital of Changsha	Changsha, Hunan, China	Screening and enrollment	the H-REPLACE Investigators
Guiyun	Ruan		MM	The Third Hospital of Changsha	Changsha, Hunan, China	Screening and enrollment	the H-REPLACE Investigators
Peng	Liu		MM	The Central Hospital of Shaoyang Shaoyang	Shaoyang, Hunan, China	Screening and enrollment	the H-REPLACE Investigators
Canxiang	Luo		MM	The Central Hospital of Shaoyang Shaoyang	Shaoyang, Hunan, China	Screening and enrollment	the H-REPLACE Investigators
Lin	He		MM	The Central Hospital of Shaoyang Shaoyang	Shaoyang, Hunan, China	Screening and enrollment	the H-REPLACE Investigators
Li	Deng		MM	The Central Hospital of Shaoyang Shaoyang	Shaoyang, Hunan, China	Screening and enrollment	the H-REPLACE Investigators
Shayan	Sun		MM	The Central Hospital of Shaoyang Shaoyang	Shaoyang, Hunan, China	Screening and enrollment	the H-REPLACE Investigators
Shuo	Yang		MM	The Central Hospital of Shaoyang Shaoyang	Shaoyang, Hunan, China	Screening and enrollment	the H-REPLACE Investigators
Sha	Wang		MM	The Central Hospital of Shaoyang Shaoyang	Shaoyang, Hunan, China	Screening and enrollment	the H-REPLACE Investigators
Songbing	Long		MM	The Central Hospital of Shaoyang Shaoyang	Shaoyang, Hunan, China	Screening and enrollment	the H-REPLACE Investigators

Supplemental Online Content: Nonauthor Collaborators
*First name, last name, and suffix (if applicable) are required and will appear in PubMed.

***First Name and Middle Initial(s)**	***Last Name**	***Suffix (eg, Jr, III)**	Academic Degrees	Institution	Location (city, state/province, country)	Role or Contribution, eg, chair, principal investigator	Group (if more than 1 Group listed in the byline) and/or Subgroup (eg, Steering Committee)
Yizhu	Duan		MM	The Central Hospital of Shaoyang Shaoyang	Shaoyang, Hunan, China	Screening and enrollment	the H-REPLACE Investigators
Jin	Luo		MM	The Central Hospital of Shaoyang Shaoyang	Shaoyang, Hunan, China	Screening and enrollment	the H-REPLACE Investigators
Zhengjie	Xue		MM	The Central Hospital of Shaoyang Shaoyang	Shaoyang, Hunan, China	Screening and enrollment	the H-REPLACE Investigators
Luzhu	Chen		MM	The Central Hospital of Shaoyang Shaoyang	Shaoyang, Hunan, China	Screening and enrollment	the H-REPLACE Investigators
Caiyan	Guo		MM	The Central Hospital of Shaoyang Shaoyang	Shaoyang, Hunan, China	Screening and enrollment	the H-REPLACE Investigators
Yongjun	Yin		MM	The Central Hospital of Shaoyang Shaoyang	Shaoyang, Hunan, China	Screening and enrollment	the H-REPLACE Investigators
Yanbo	Wang		MM	The Central Hospital of Shaoyang Shaoyang	Shaoyang, Hunan, China	Screening and enrollment	the H-REPLACE Investigators
Fan	Ouyang		MD	Zhuzhou Central Hospital	Zhuzhou, Hunan, China	Sub-investigator	the H-REPLACE Investigators
Shuhong	Guo		MD	Zhuzhou Central Hospital	Zhuzhou, Hunan, China	Screening and enrollment	the H-REPLACE Investigators
Weiqing	Liu		MM	Zhuzhou Central Hospital	Zhuzhou, Hunan, China	Screening and enrollment	the H-REPLACE Investigators
Chunfeng	Liao		MM	The First Hospital of Changsha	Changsha, Hunan, China	Screening and enrollment	the H-REPLACE Investigators
Dongsheng	Lin		MM	The First Hospital of Changsha	Changsha, Hunan, China	Screening and enrollment	the H-REPLACE Investigators
Huijuan	Chen		MM	The First Hospital of Changsha	Changsha, Hunan, China	Screening and enrollment	the H-REPLACE Investigators
Lixia	Ma		MM	The First Hospital of Changsha	Changsha, Hunan, China	Screening and enrollment	the H-REPLACE Investigators
Hui	Luo		MD	The First Hospital of Changsha	Changsha, Hunan, China	Screening and enrollment	the H-REPLACE Investigators
Jianfeng	Wu		MM	The Second Affiliated Hospital of University of South China	Hengyang, Hunan, China	data collection	the H-REPLACE Investigators
Chao	Yang		MM	The Second Affiliated Hospital of University of South China	Hengyang, Hunan, China	Screening and enrollment	the H-REPLACE Investigators

Supplemental Online Content: Nonauthor Collaborators
*First name, last name, and suffix (if applicable) are required and will appear in PubMed.

***First Name and Middle Initial(s)**	***Last Name**	***Suffix (eg, Jr, III)**	Academic Degrees	Institution	Location (city, state/province, country)	Role or Contribution, eg, chair, principal investigator	Group (if more than 1 Group listed in the byline) and/or Subgroup (eg, Steering Committee)
Yang	Liu		MM	The Second Affiliated Hospital of University of South China	Hengyang, Hunan, China	Screening and enrollment	the H-REPLACE Investigators
Yingnan	Qin		MM	The Second Affiliated Hospital of University of South China	Hengyang, Hunan, China	Screening and enrollment	the H-REPLACE Investigators
Fangyao	Liu		MM	The Second Affiliated Hospital of University of South China	Hengyang, Hunan, China	Screening and enrollment	the H-REPLACE Investigators
Jianqiang	Xu		MM	The Second Affiliated Hospital of University of South China	Hengyang, Hunan, China	Screening and enrollment	the H-REPLACE Investigators
Juan	Tang		MM	The Second Affiliated Hospital of University of South China	Hengyang, Hunan, China	Screening and enrollment	the H-REPLACE Investigators
Yixin	Tang		MD	The First Affiliated Hospital of University of South China	Hengyang, Hunan, China	data collection	the H-REPLACE Investigators
Hengjing	Hu		MM	The First Affiliated Hospital of University of South China	Hengyang, Hunan, China	Screening and enrollment	the H-REPLACE Investigators
Jiming	Zhou		MM	The First Affiliated Hospital of University of South China	Hengyang, Hunan, China	Screening and enrollment	the H-REPLACE Investigators
Xing	Xiang		MM	The First People's Hospital of Huaihua	Huaihua, Hunan,China	Screening and enrollment	the H-REPLACE Investigators
Jingyong	Yang		MM	The First People's Hospital of Huaihua	Huaihua, Hunan,China	Screening and enrollment	the H-REPLACE Investigators
Tingting	Deng		MM	The First People's Hospital of Huaihua	Huaihua, Hunan,China	Screening and enrollment	the H-REPLACE Investigators
Ping	Xiao		MM	The First People's Hospital of Huaihua	Huaihua, Hunan,China	Screening and enrollment	the H-REPLACE Investigators
Jun	Liu		MM	The First People's Hospital of Huaihua	Huaihua, Hunan,China	Screening and enrollment	the H-REPLACE Investigators
Shenyi	Guo		MM	The First People's Hospital of Huaihua	Huaihua, Hunan,China	Screening and enrollment	the H-REPLACE Investigators
Wenjie	Liu		MM	The First People's Hospital of Huaihua	Huaihua, Hunan,China	Screening and enrollment	the H-REPLACE Investigators
Xing	Zhang		MM	The First People's Hospital of Huaihua	Huaihua, Hunan,China	Screening and enrollment	the H-REPLACE Investigators

Supplemental Online Content: Nonauthor Collaborators
*First name, last name, and suffix (if applicable) are required and will appear in PubMed.

***First Name and Middle Initial(s)**	***Last Name**	***Suffix (eg, Jr, III)**	Academic Degrees	Institution	Location (city, state/province, country)	Role or Contribution, eg, chair, principal investigator	Group (if more than 1 Group listed in the byline) and/or Subgroup (eg, Steering Committee)
Zhihong	Li		MM	The First People's Hospital of Huaihua	Huaihua, Hunan,China	Screening and enrollment	the H-REPLACE Investigators
Renhua	Zeng		MM	The First People's Hospital of Huaihua	Huaihua, Hunan,China	Screening and enrollment	the H-REPLACE Investigators
Zhen	Zhou		MD	Yongzhou First People's Hospital	Yongzhou, Hunan, China	Sub-investigator	the H-REPLACE Investigators
Jianhong	Bao		MM	Yongzhou First People's Hospital	Yongzhou, Hunan, China	data collection	the H-REPLACE Investigators
Ye	Zhou		MM	Yongzhou First People's Hospital	Yongzhou, Hunan, China	Screening and enrollment	the H-REPLACE Investigators
Xiaojuan	Wang		MM	Yongzhou First People's Hospital	Yongzhou, Hunan, China	Screening and enrollment	the H-REPLACE Investigators
He	Huang		MD	Xiangtan Central Hospital	Xiangtan, Hunan, China	Sub-investigator	the H-REPLACE Investigators
Yunlong	Zhu		MD	Xiangtan Central Hospital	Xiangtan, Hunan, China	data collection	the H-REPLACE Investigators
Wenjiao	Zhao		MM	Xiangtan Central Hospital	Xiangtan, Hunan, China	Screening and enrollment	the H-REPLACE Investigators
Xianming	Wu		MM	Yiyang Central Hospital	Yiyang, Hunan,China	Sub-investigator	the H-REPLACE Investigators
Ming	Cai		MM	Yiyang Central Hospital	Yiyang, Hunan,China	data collection	the H-REPLACE Investigators
Fan	Liu		MM	Yiyang Central Hospital	Yiyang, Hunan,China	Screening and enrollment	the H-REPLACE Investigators
Luping	Jiang		MD	Changsha Central Hospital	Changsha, Hunan, China	Sub-investigator	the H-REPLACE Investigators
Zehua	Li		MM	Changsha Central Hospital	Changsha, Hunan, China	data collection	the H-REPLACE Investigators
Songquan	Peng		MM	Changsha Central Hospital	Changsha, Hunan, China	Screening and enrollment	the H-REPLACE Investigators
Qiao	Jin		MM	Changsha Central Hospital	Changsha, Hunan, China	Screening and enrollment	the H-REPLACE Investigators
Chun	Chen		MM	Changsha Central Hospital	Changsha, Hunan, China	Screening and enrollment	the H-REPLACE Investigators
Lu	Hu		MM	Changsha Central Hospital	Changsha, Hunan, China	Screening and enrollment	the H-REPLACE Investigators
Ting	Zhou		MM	Changsha Central Hospital	Changsha, Hunan, China	Screening and enrollment	the H-REPLACE Investigators
Pengcui	Wu		MM	Changsha Central Hospital	Changsha, Hunan, China	Screening and enrollment	the H-REPLACE Investigators

*First name, last name, and suffix (if applicable) are required and will appear in PubMed.

***First Name and Middle Initial(s)**	***Last Name**	***Suffix (eg, Jr, III)**	Academic Degrees	Institution	Location (city, state/province, country)	Role or Contribution, eg, chair, principal investigator	Group (if more than 1 Group listed in the byline) and/or Subgroup (eg, Steering Committee)
Xiao	Long		MM	Changsha Central Hospital	Changsha, Hunan, China	Screening and enrollment	the H-REPLACE Investigators
Yuyan	Huang		MM	Changsha Central Hospital	Changsha, Hunan, China	Screening and enrollment	the H-REPLACE Investigators
Hebin	Xie		MM	Changsha Central Hospital	Changsha, Hunan, China	Screening and enrollment	the H-REPLACE Investigators
Tao	Yang		MM	Changsha Central Hospital	Changsha, Hunan, China	Screening and enrollment	the H-REPLACE Investigators
Shao	Ouyang		MM	The Second Affiliated Hospital of University of South China	Hengyang, Hunan, China	data collection	the H-REPLACE Investigators
Xiang	Liu		MD	The Second People's Hospital of Hunan Province	Changsha, Hunan, China	Screening and enrollment	the H-REPLACE Investigators
Song	Hu		MM	The Second People's Hospital of Hunan Province	Changsha, Hunan, China	Screening and enrollment	the H-REPLACE Investigators
Zhenhua	Xing		MM	The Second People's Hospital of Hunan Province	Changsha, Hunan, China	Screening and enrollment	the H-REPLACE Investigators
Jianping	Zeng		MD	Xiangtan Central Hospital	Xiangtan, Hunan, China	Member of Endpoint Adjudication Committee	the H-REPLACE Investigators
Yi	Zhang		MD	Hunan Provincial People's Hospital, The First Affiliated Hospital of Hunan Normal University	Changsha, Hunan, China	Member of Endpoint Adjudication Committee	the H-REPLACE Investigators
Xiang	Liu		MD	The Second People's Hospital of Hunan Province	Changsha, Hunan, China	Member of Endpoint Adjudication Committee	the H-REPLACE Investigators
