# Supplementary material for: Effect of Rivaroxaban vs Enoxaparin on Major Cardiac Adverse Events and Bleeding Risk in the Acute Phase of Acute Coronary Syndrome: The H-REPLACE Randomized Equivalence and Noninferiority Trial
Source: JAMA Netw Open. 2023 Feb 10;6(2):e2255709. doi: 10.1001/jamanetworkopen.2022.55709 (PMC9918885; doi:10.1001/jamanetworkopen.2022.55709)
Supplement: Supplement 4. — Data Sharing Statement [file jamanetwopen-e2255709-s004.pdf]

Data Sharing Statement

Zhou. Effect of Rivaroxaban vs Enoxaparin on Major Cardiac Adverse Events and Bleeding Risk in the Acute Phase of Acute Coronary Syndrome. *JAMA Netw Open*. Published February 10, 2023. doi:10.1001/jamanetworkopen.2022.55709

Data
Data available: Yes
Data types: Data dictionary
How to access data: zhoushenghua@csu.edu.cn
When available: With publication

Supporting Documents
Document types: Statistical/analytic code, Informed consent form
How to access documents: zhoushenghua@csu.edu.cn
When available: With publication

Additional Information
Who can access the data: researchers whose proposed use of the data has been approved.
Types of analyses: For academic purpose.
Mechanisms of data availability: with a signed data access agreement
